# Species richness of bat flies and their associations with host bats in a subtropical East Asian region

**DOI:** 10.1186/s13071-023-05663-x

**Published:** 2023-01-27

**Authors:** Emily Shui Kei Poon, Guoling Chen, Hiu Yu Tsang, Chung Tong Shek, Wing Chi Tsui, Huabin Zhao, Benoit Guénard, Simon Yung Wa Sin

**Affiliations:** 1 School of Biological Sciences, The University of Hong Kong, Pok Fu Lam Road, Hong Kong, China; 2 Agriculture, Fisheries and Conservation Department (AFCD), Hong Kong SAR Government, Hong Kong, China; 3 Department of Ecology, College of Life Sciences, Wuhan University, Wuhan, 430072 Hubei China

**Keywords:** Bat parasites, Ectoparasite, Host specificity, Host-parasite coevolution, Nycteribiidae, Streblidae

## Abstract

**Background:**

Understanding the interactions between bat flies and host bats offer us fundamental insights into the coevolutionary and ecological processes in host-parasite relationships. Here, we investigated the identities, host specificity, and patterns of host association of bat flies in a subtropical region in East Asia, which is an understudied region for bat fly research.

**Methods:**

We used both morphological characteristics and DNA barcoding to identify the bat fly species found on 11 cavernicolous bat species from five bat families inhabiting Hong Kong. We first determined the phylogenetic relationships among bat fly species. Then, we elucidated the patterns of bat-bat fly associations and calculated the host specificity of each bat fly species. Furthermore, we assembled the mitogenomes of three bat fly species from two families (Nycteribiidae and Streblidae) to contribute to the limited bat fly genetic resources available.

**Results:**

We examined 641 individuals of bat flies and found 20 species, of which many appeared to be new to science. Species of Nycteribiidae included five *Nycteribia* spp., three *Penicillidia* spp., two *Phthiridium* spp., one *Basilia* sp., and one species from a hitherto unknown genus, whereas Streblidae included *Brachytarsina amboinensis*, three *Raymondia* spp., and four additional *Brachytarsina* spp. Our bat-bat fly association network shows that certain closely related bat flies within Nycteribiidae and Streblidae only parasitized host bat species that are phylogenetically more closely related. For example, congenerics of *Raymondia* only parasitized hosts in *Rhinolophus* and *Hipposideros*, which are in two closely related families in Rhinolophoidea, but not other distantly related co-roosting species. A wide spectrum of host specificity of these bat fly species was also revealed, with some bat fly species being strictly monoxenous, e.g. nycteribiid *Nycteribia* sp. A, *Phthiridium* sp. A, and streblid *Raymondia* sp. A, while streblid *B. amboinensis* is polyxenous.

**Conclusions:**

The bat fly diversity and specificity uncovered in this study have shed light on the complex bat-bat fly ecology in the region, but more bat-parasite association studies are still needed in East Asian regions like China as a huge number of unknown species likely exists. We highly recommend the use of DNA barcoding to support morphological identification to reveal accurate host-ectoparasite relationships for future studies.

**Graphical Abstract:**

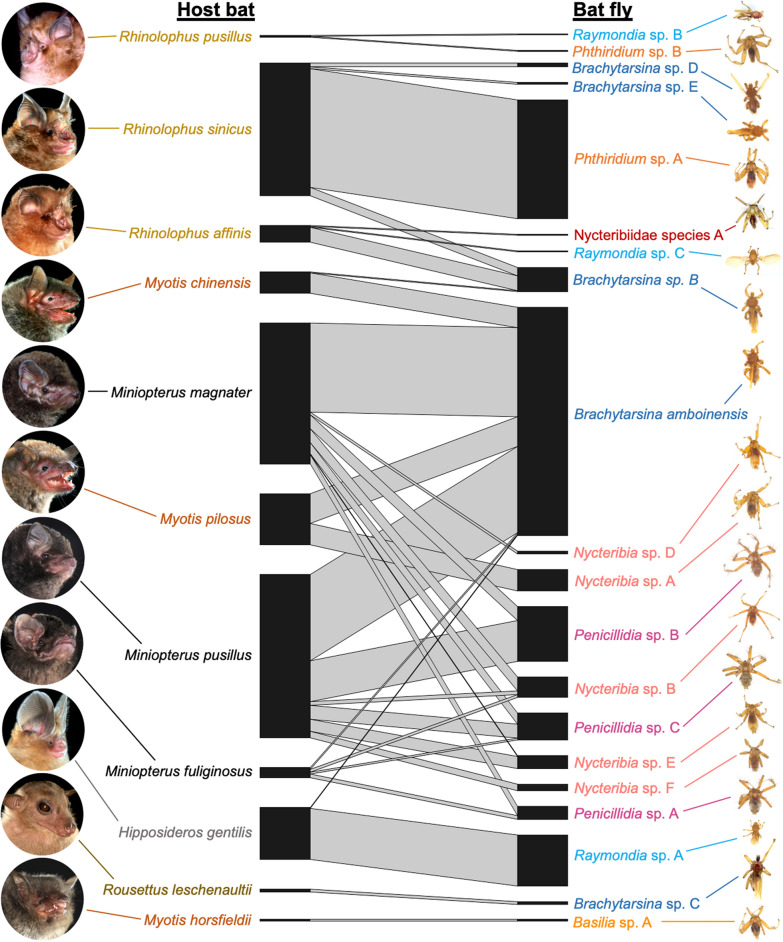

**Supplementary Information:**

The online version contains supplementary material available at 10.1186/s13071-023-05663-x.

## Background

Interactions between parasites and their hosts are one of the most striking coevolutionary arms races [1]. Both parasites and hosts exert selective pressures over each other, leading to reciprocal adaptations over time [2, 3]. Hippoboscoidea superfamily (Diptera) members have evolved to become obligate parasites with adaptations for feeding on the blood of vertebrates. Members of Hippoboscoidea belong to one of four families, Glossinidae, Hippoboscidae, Nycteribiidae, and Streblidae [4], with the latter two being ectoparasites exclusively associated with bats (Chiroptera). Co-evolving with bats for more than 15 million years [5], bat flies are highly adapted to live on the fur and wing or tail membranes of bats; they are the most prevalent ectoparasites on bats worldwide. Some streblids develop fully functional wings after pupation and can fly to search for hosts, while others that possess rudimentary or no wings and nycteribiids, which are all wingless, can only rely on crawling behaviors to reach their hosts [4]. Approximately 230 species in 33 genera from Streblidae and 280 species in 11 genera from Nycteribiidae have been recorded to date [6]. Nycteribiid and streblid species are not equally distributed among global regions [7, 8]. Nearly 70% of streblid species are distributed within the New World tropics or subtropics, and relatively few species occur exclusively in temperate zones, whereas about 80% of nycteribiid species occur in the Old World tropics or subtropics [4]. The most recent phylogenetic study showed that Nycteribiidae is monophyletic but Streblidae is a paraphyletic group, which comprises the monophyletic New World clade and the paraphyletic Old World clade, which clusters with Nycteribiidae [9]. The diversity of bat flies and their unique relationships with host bats make them an inviting model system for studying several ecological and evolutionary questions in the past decades, including systematics and biogeography, intra- or interspecific transmission of pathogens, and host-ectoparasite interaction dynamics [10].

Dynamics of bat-bat fly interactions are complex and influenced by the interplay of numerous biotic and abiotic variables. For example, roosting site preference and roosting behaviors of bats can affect the transmissibility of their bat flies. Bat populations roosting in permanent and protective structures (e.g. caves, water tunnels, and abandoned mines) were found to experience heavier bat fly parasitism than those more temporary roosting sites, such as foliage, tree trunks, and other open spots [11–13]. Bats that used more caves to roost and remained in caves for longer times were more likely to be infested by ectoparasites [14]. Moreover, the morphophysiological traits of bat hosts, such as sex, body conditions, and reproductive cycle, were found to affect the host preference of bat flies or level of bat fly parasitism [15–17]. The life histories and morphological traits of ectoparasites are also the key determinants in their dispersal capability [18, 19]. Vegetation type and seasonality could also promote changes in the bat-bat fly interaction network in a region [20]. Recent studies demonstrated that bat flies generally exhibited high host specificity and often formed distinct species assemblages on their host bat species [21]. Individual bat species typically support one to five species of bat flies [22] with phylogenetically distant bat fly species co-occurring on a similar host occupying different areas of the host's body to reduce competition for space and resources [23].

Although the distribution, species richness, and host-ectoparasite associations of bat flies in the Americas, Africa, and Southeast Asia have been relatively well documented over the years, knowledge on bat flies in East Asia, especially China, is largely lacking [24, 25]. Considering the importance of this geographic region in multiple outbreaks of severe zoonotic diseases linked to bats [26, 27], understanding the diversity of bat flies and their association to bats in this region appears especially urgent from ecological and public health perspectives. Bats are well known to be important natural reservoirs of zoonotic pathogens [28], and bat flies were proposed to function as vectors for certain bat-associated pathogens, in which some also possess high zoonotic potential [29, 30].

One difficulty of bat fly research for understudied regions is the identification of specimens based on a few taxonomic publications developed more than half a century ago [31–34] from a limited and incomplete pool of species, a problem more widely known as the Linnean shortfall [35, 36]. Occurrences of cryptic species and phenotypic plasticity represent additional difficulties to morphologically distinguish bat fly species [33, 37]. DNA barcoding was thus recommended as a potential approach to reliably separate bat fly species [13, 37]. In this study, we sought to determine the species richness, phylogenetic relationships, and host association patterns of bat flies found on 11 cavernicolous bat species occurring in China, using both morphological and DNA barcoding approaches. Hong Kong, located in the subtropical region of East Asia, is home to 25 bat species recorded to date [38]; however, the bat flies parasitizing many of these bats in the region remain undocumented. Specifically, we aimed to (i) use morphological characteristics and DNA barcoding to distinguish and identify the bat fly species found on 11 cavernicolous bat species in Hong Kong; (ii) determine the phylogenetic relationships among the bat fly species; (iii) elucidate the patterns of bat-bat fly association and evaluate the degrees of specificity of each bat fly species to their host species. In addition, we also (iv) assemble the mitogenomes of one nycteribiid and two streblid species from different genera to enrich the limited genetic resources available from bat flies for assisting primer design and species identification in future studies. Our findings will provide new knowledge on the species richness of bat flies and the bat-bat fly association network, reflecting the ecological and coevolutionary relationships among bat and bat flies, in an understudied region of unneglectable public health concern.

## Methods

### Specimen collection

Bats roosting in abandoned mines, water tunnels, and culverts in Hong Kong were captured by hand-held hoop nets during 2018–2022. To avoid inter-host contamination of bat flies, only one bat was kept in each sterilized cloth bag. Each bat was identified to species by morphology [39]. *Miniopterus magnater* and *Miniopterus fuliginosus* were morphologically similar, so we used a sterilized wing punch tool to collect 5-mm tissues from the wing membranes for DNA barcoding to confirm their species identity [40]. Bat flies were collected from each bat using a blunt-end forceps. All bats were released back into the wild after sample collection. All samples were immediately preserved in absolute ethanol in the field and stored at −20 °C on the same day until microscopic examination or DNA extraction.

### Microscopic examination and DNA barcoding

We examined and photographed all bat fly specimens using a compound microscope (Leica M205 C, Wetzlar, Germany). We separated the bat flies based on their sex and identified the species in each sex based on their morphologies [31–33]. A subset of the specimens from each morphospecies in each sex of bat fly (120 samples) and all *Miniopterus* tissues were then proceeded to DNA extraction using the E.Z.N.A. Tissue DNA Kit (Omega bio-tek, Norcross, USA). We DNA barcoded each sample by using the primer pair LCO1490 (forward primer: 5'GGTCAACAAATCATAAAGATATTGG 3') and HCO2198 (reverse primer: 5'TAAACTTCAGGGTGACCAAAAAATCA3') to amplify a 658 bp DNA fragment from the mitochondrial cytochrome c oxidase subunit I gene (*COI*) [41]. Each DNA barcoding PCR was performed in 30 μl reaction, containing 6 μl 5X GoTaq Flexi Buffer, 0.6 μl 10 mM dNTP Mix, 3.6 μl 25 mM MgCl_2_, 0.15 μl 5 U/μl GoTaq G2 Flexi DNA Polymerase (Promega, Madison, USA), 1 μl extracted DNA, 0.6 μl of each primer, 6 μl 10% DMSO (Sigma, Burlington, MA, USA), and ultrapure water. Thermal cycling condition of the PCR was 95 °C for 2 min; 35 cycles of 95 °C for 30 s, 56 °C for 30 s and 72 °C for 1 min; and final extension at 72 °C for 5 min. The target sizes of PCR products were confirmed by gel electrophoresis, and the PCR products were sequenced by BGI (Shenzhen, China).

### Phylogenetic analysis

Seventy-one *COI* sequences of bat flies were used for phylogenetic analysis, in which 20 and 51 sequences were obtained from this study and Genbank, respectively. We aligned the sequences with Clustal W [42] algorithm using MEGA 6.06 [43]. We used ModelFinder in IQ-Tree v2.20 [44] to find the best-fit model of nucleotide substitution, which was the General Time-Reversible (GTR) model with gamma-shaped (G) distribution across sites and invariable sites (I). Maximum likelihood (ML) and Bayesian inference (BI) methods were used for the reconstruction of phylogenetic trees. The ML tree was run for 1000 bootstrap replications using IQ-Tree v2.20 [44]. A Markov chain Monte Carlo (MCMC) search was initiated with random trees and run for 2,000,000 generations using MrBayes v3.2.7 [45], with a sampling frequency of every 1000 generations, and the first 25% of samples were discarded as burn-in. Trees were visualized using FigTree v1.4.4 [46]. We calculated the pairwise p-distances using MEGA 6.06 [43]. Identities of bat fly species with distinct *COI* sequences (> 2% difference) [47] were cross-checked with the morphological descriptions and illustrations presented in the literature [31–33].

### Host specificity analysis

We estimated the host specificity of each bat fly species by calculating (i) number of bat fly-infested bats of a bat species (Nb); (ii) number of bat flies of a bat fly species found on the bats of a bat species (Ne); (iii) number of bats of a bat species infested with a particular bat fly species (Nib); (iv) specificity index (SI), which is the percentage of total number of bat flies of a single species found on the host bats of a bat species, i.e. SI of bat fly species A = Ne of species A/total number of species A individuals found on all bats × 100 [48].

### Mitogenome assembly and annotation

DNA library of 350-bp insert size was prepared for each of the three bat fly species (i.e. nycteribiid *Phthiridium* sp. A and streblids *Brachytarsina amboinensis* and *Raymondia* sp. A). The libraries were sequenced on an Illumina NovaSeq instrument (PE 150 bp reads) for 8 G per sample at Novogene. We filtered the sequencing reads using fastp [49] and evaluated the quality of filtered reads with FASTQC [50]. Then, we assembled the mitogenome by MIRA v4.0 [51] and MITOBIM.PL v1.6 [52], using a house fly mitogenome (*Musca domestica*, accession no. NC024855.1) as a reference. We annotated these three mitogenomes using MITOS [53] and GeSeq [54]; then, we checked the annotation manually based on the annotations from the mitogenomes of three close relatives (NC024855.1: *Musca domestica*, MK896866.1: *Paratrichobius longicrus*, and MK896865.1: *Paradyschiria parvula*). The protein translation was corrected according to the BLAST results and the annotations of these three close relatives. The final mitogenome annotations were visualized using OGDRAW [55].

## Results

### Bat fly identification

**Fig. 1:**
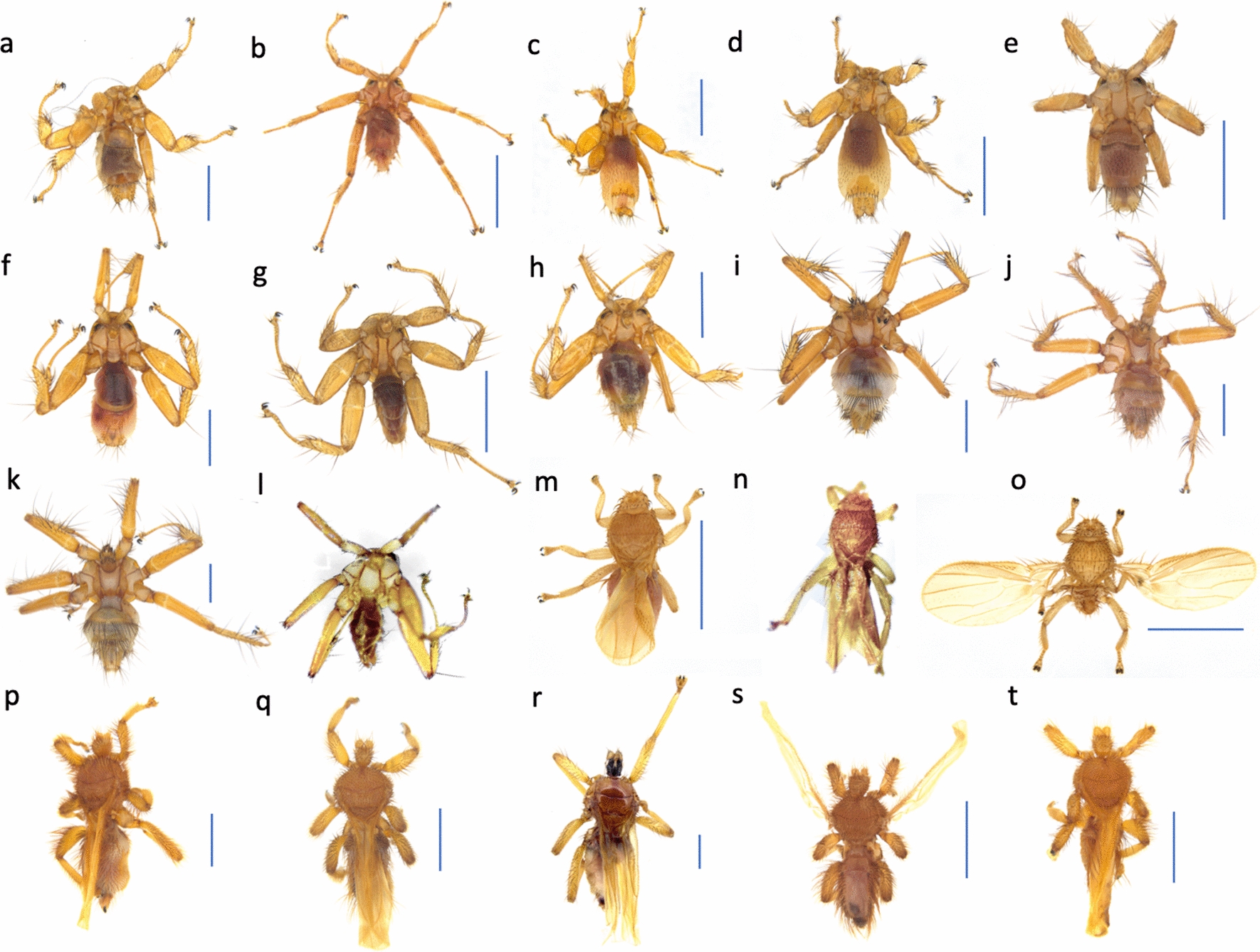
Images of the dorsal views of female bat flies, unless otherwise specified. Bat flies labeled a-l and m-t belong to Nycteribiidae and Streblidae, respectively. **a** *Nycteribia* sp. A; **b** *Nycteribia* sp. B; **c** *Nycteribia* sp. D; **d** *Nycteribia* sp. E; **e** *Nycteribia* sp. F; **f** *Phthiridium* sp. A; **g** *Phthiridium* sp. B, male only; **h** *Basilia* sp. A; **i** *Penicillidia* sp. A; **j** *Penicillidia* sp. B; **k** *Penicillidia* sp. C; **l** Nycteribiidae species A, male only, scale bar unavailable; **m** *Raymondia* sp. A; **n** *Raymondia* sp. B, male only, scale bar unavailable; **o** *Raymondia* sp. C, image shown was a protease-digested specimen and abdomen was contracted; **p** *Brachytarsina amboinensis*; **q** *Brachytarsina* sp. B; **r** *Brachytarsina* sp. C; **s** *Brachytarsina* sp. D, male; and **t** *Brachytarsina* sp. E, male only. Blue scale bar = 1 mm. Refer to Additional file 1: Figs. S1–S20 for the corresponding images of the ventral views of females and the dorsal and ventral views of males

We collected 641 bat flies from 271 bats representing 11 bat species in five bat families, i.e. *Rhinolophus sinicus*, *R. affinis*, and *R. pusillus* in Rhinolophidae; *Myotis chinensis*, *M. pilosus*, and *M. horsfieldii* in Vespertilionidae; *Miniopterus magnater*, *M. fuliginosus*, and *M. pusillus* in Miniopteridae; *Hipposideros gentilis* in Hipposideridae; and *Rousettus leschenaultii* in Pteropodidae (Table 1). We found 20 bat fly species, with 12 and eight species belonging to Nycteribiidae and Streblidae, respectively (Fig. 1 and Additional file 1: Figs. S1-S20). Regarding the nycteribiids, there were five *Nycteribia* spp., three *Penicillidia* spp., two *Phthiridium* spp., one *Basilia* sp., and one from a hitherto unknown genus (Nycteribiidae species A) (Fig. 1 and Additional file 1: Figs. S1-S12). These nycteribiids all belong to the subfamily Nycteribiinae. For the streblids, we identified *B. amboinensis* (Rondani 1878), three *Raymondia* spp. [31, 32], and four *Brachytarsina* spp. (Fig. 1 and Additional file 1: Figs. S13-S20). They all belong to the subfamily Brachytarsininae. Species identified at the genus or family levels could be classified to neither species level based on *COI* nor morphology. Both sexes of each species were found except for five species (Table 1). All streblids found in this study possessed functional wings.

**Table 1:** Host-ectoparasite association network

Family of bat species	Bat species	Nb	Bat fly taxon	Family of bat fly taxon	Ne	Number of female flies	% of female flies	Number of male flies	% of male flies	Number of flies with unknown sex	Nib	SI
Rhinolophidae	*Rhinolophus sinicus*	58	*Brachytarsina* sp. B	Streblidae	9	3	33	6	67	0	9	34.62
			*Brachytarsina* sp. D	Streblidae	4	1	25	3	75	0	4	100.00
			*Brachytarsina* sp. E	Streblidae	2	0	0	2	100	0	2	100.00
			*Phthiridium* sp. A	Nycteribiidae	128	73	57	55	43	0	50	100.00
	*Rhinolophus affinis*	8	*Brachytarsina* sp. B	Streblidae	16	3	19	13	81	0	6	61.54
			*Raymondia* sp. C	Streblidae	1	1	100	0	0	0	1	50.00
			Nycteribiidae sp. A	Nycteribiidae	1	0	0	1	100	0	1	100.00
	*Rhinolophus pusillus*	2	*Raymondia* sp. B	Streblidae	1	0	0	1	100	0	1	50.00
			*Phthiridium* sp. B	Nycteribiidae	1	0	0	1	100	0	1	100.00
Vespertilionidae	*Myotis chinensis*	13	*Brachytarsina amboinensis*	Streblidae	22	9	41	13	59	0	12	8.94
			*Brachytarsina* sp. B	Streblidae	1	0	0	1	100	0	1	3.85
	*Myotis pilosus*	37	*B. amboinensis*	Streblidae	32	18	56	14	44	0	27	13.01
			*Nycteribia* sp. A	Nycteribiidae	23	10	43	13	57	0	15	100.00
	*Myotis horsfieldii*	2	*Basilia* sp. A	Nycteribiidae	2	2	100	0	0	0	2	100.00
Miniopteridae	*Miniopterus magnater*	49	*B. amboinensis*	Streblidae	96	58	60	37	39	1	39	39.02
			*Nycteribia* sp. B	Nycteribiidae	15	7	47	8	53	0	14	68.18
			*Nycteribia* sp. D	Nycteribiidae	3	2	67	1	33	0	3	100.00
			*Nycteribia* sp. E	Nycteribiidae	1	1	100	0	0	0	1	7.14
			*Penicillidia* sp. A	Nycteribiidae	10	3	30	7	70	0	9	71.43
			*Penicillidia* sp. B	Nycteribiidae	15	11	73	4	27	0	11	25.42
			*Penicillidia* sp. C	Nycteribiidae	12	4	33	8	67	0	11	41.38
	*Miniopterus fuliginosus*	4	*B. amboinensis*	Streblidae	2	2	100	0	0	0	1	0.81
			*Nycteribia* sp. B	Nycteribiidae	3	1	33	2	67	0	2	13.64
			*Penicillidia* sp. A	Nycteribiidae	4	2	50	2	50	0	3	28.57
			*Penicillidia* sp. C	Nycteribiidae	2	2	100	0	0	0	2	6.90
	*Miniopterus pusillus*	72	*B. amboinensis*	Streblidae	93	56	60	35	38	2	50	37.80
			*Nycteribia* sp. B	Nycteribiidae	4	3	75	1	25	0	4	18.18
			*Nycteribia* sp. E	Nycteribiidae	13	5	38	8	62	0	10	92.86
			*Nycteribia* sp. F	Nycteribiidae	7	5	71	2	29	0	7	100.00
			*Penicillidia* sp. B	Nycteribiidae	44	18	41	26	59	0	31	74.58
			*Penicillidia* sp. C	Nycteribiidae	15	7	47	8	53	0	11	51.72
Hipposideridae	*Hipposideros gentilis*	25	*B. amboinensis*	Streblidae	1	0	0	1	100	0	1	0.41
			*Raymondia* sp. A	Streblidae	55	25	45	28	51	2	24	100.00
Pteropodidae	*Rousettus leschenaultii*	1	*Brachytarsina* sp. C	Streblidae	3	2	67	1	33	0	1	100.00

*Nb* number of infested bats of a bat species, *Ne* number of bat flies of a bat fly species found on the bats of a bat species, *Nib* number of bats of a bat species infested with a particular bat fly species, *SI* specificity index, percentage of total number of bat flies of a single species found on the host bats of a bat species

### Phylogenetic relationships among the bat fly species

**Fig. 2:**
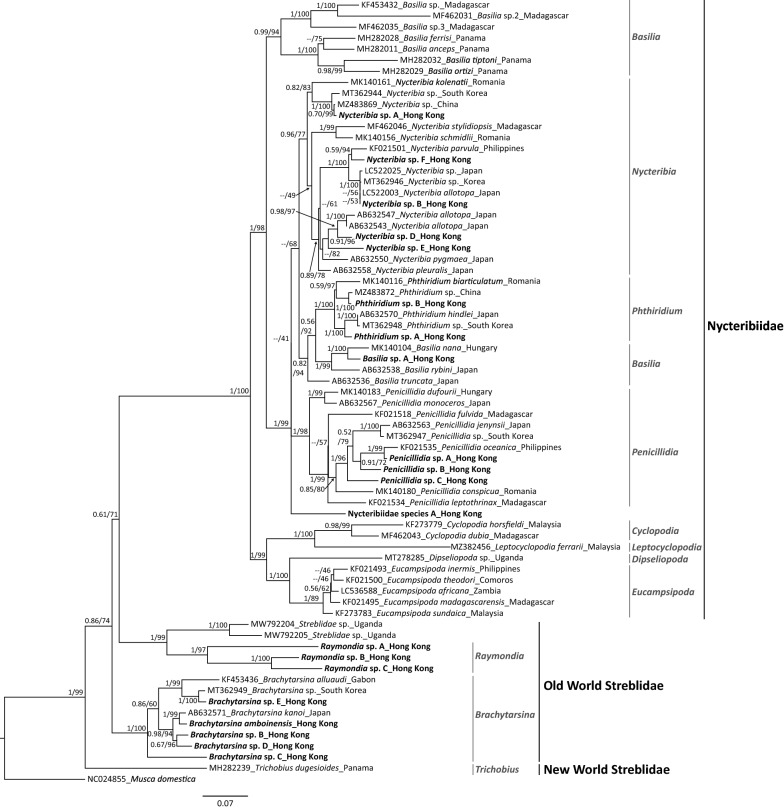
Phylogenetic relationships among bat fly species. The phylogenetic tree was inferred by maximum likelihood (ML) and Bayesian inference (BI) methods based on *COI* gene (658 bp). The values at each node represent the Bayesian posterior probability and the ML bootstrap value. A dash (–) indicates that the topologies of the ML tree and BI tree do not coincide at that branch and only the ML bootstrap value is shown. The vertical lines on the right indicate the genera or families of these bat fly species

Among the *Nycteribia* spp. identified, *Nycteribia* sp. A was the most distantly related to the other four *Nycteribia* spp., and it grouped with a *Nycteribia* specimen known from China (p-distance = 0.3%) (Fig. 2; refer to Additional file 1: Fig. S21 for the phylogenetic trees using more conspecifics for each identified species). *Nycteribia* sp. B clustered with a *Nycteribia* specimen from Japan (*P*-distance = 0.3%) identified as *Nycteribia allotopa*. In a separate clade, another two sequences also identified as *N. allotopa* clustered with *Nycteribia* sp. D (*P*-distance = 2.7–3.7%) and sp. E (*P*-distance = 5.3–5.5%). *Nycteribia* sp. E resembled *N. allotopa* the most among the species of *Nycteribia* from Hong Kong according to the taxonomic information within Speiser (1901) [33]. *Nycteribia* sp. F was closely related to a specimen identified as *Nycteribia parvula* from the Philippines (*P*-distance = 3.2%) (Fig. 2). *Phthiridium* sp. A clustered with a specimen identified as *Phthiridium hindlei* from Japan (*P*-distance = 2.4%) and a specimen identified as *Phthiridium* sp. from South Korea (*P*-distance = 2.7%) to form a well-supported clade. *Phthiridium* sp. B grouped with an unidentified species of *Phthiridium* from China (*P*-distance = 0.8%). In the polyphyletic genus *Basilia*, *Basilia* sp. A was closely related to *Basilia nana* identified from Hungary (*P*-distance = 4%) (Fig. 2). *Penicillidia* sp. A grouped with *Penicillidia oceanica* identified from the Philippines (*P*-distance = 2.1%) and further clustered with *Penicillidia* sp. B as a clade. *Penicillidia* sp. C was sister to this clade. Nycteribiidae species A is sister to the clades of the four aforementioned nycteribiid genera, but its exact genus identity could not be determined (Fig. 2).

All streblid species identified belong to the Old World Streblidae, which is a paraphyletic group clustered with Nycteribiidae [9] (Fig. 2). *Raymondia* sp. B and sp. C were highly divergent (*P*-distance = 7.9%), and they grouped together and formed a clade with *Raymondia* sp. A. The clade of *Raymondia* spp. was sister to a clade of two species of Streblidae from Uganda (*P*-distance = 15.4–18%; Fig. 2). These two sister clades formed a monophyletic group with Nycteribiidae instead of other Old World Streblidae. *Raymondia* sp. C highly resembled *Raymondia pagodarum* (Speiser 1900) based on morphology [31], but no *COI* sequence of *Raymondia* species was available in GenBank. *Brachytarsina* sp. E was closely related to an unidentified specimen of *Brachytarsina* sp. from South Korea (*P*-distance = 1.7%). *Brachytarsina amboinensis* grouped with *Brachytarsina kanoi* in Japan (*P*-distance = 2%) as a clade and clustered with the clade of *Brachytarsina* sp. B and sp. D (p-distance between B and D = 3%). No *COI* sequence of *B. amboinensis* was available in GenBank. *Brachytarsina* sp. B and sp. D were superficially similar to *Brachytarsina werneri* (Jobling 1951) but slight morphological differences could be observed [32]. For example, the two lateral parts of the seventh tergite present in female *B. werneri* were not observed in *Brachytarsina* sp. B and sp. D. *Brachytarsina* sp. C is the most divergent from the four aforementioned *Brachytarsina* species (Fig. 2).

### Patterns of bat-bat fly association

**Fig. 3:**
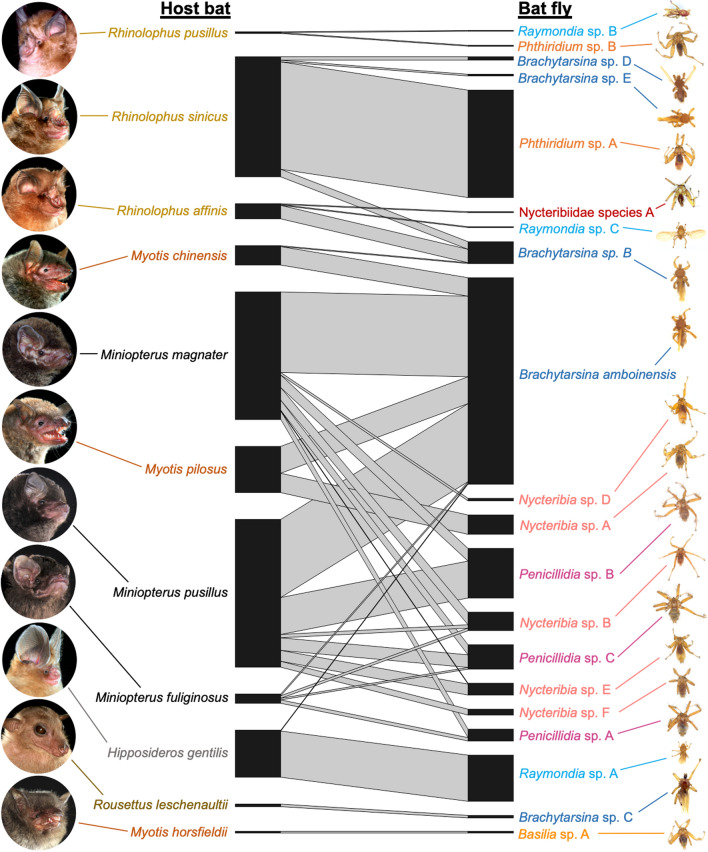
Host-ectoparasite association network. Web of interactions between bat fly species (right) and bat species (left). The width of bars is proportional to the number of bat fly individuals. Names of species that belong to the same genus are in the same color. Bat photos^©^Agriculture, Fisheries and Conservation Department

The bat fly species that exhibited the highest host specificity (SI = 100, Fig. 3, and Table 1) included nycteribiid *Phthiridium* sp. A, *Nycteribia* sp. A, *Nycteribia* sp. F, and streblid *Raymondia* sp. A. Remarkably, each of these bat fly species was highly specific to a single bat species in a different family, i.e. *Phthiridium* sp. A (*n* = 128) on *R. sinicus* in Rhinolophidae; *Raymondia* sp. A (*n* = 55) on *H. gentilis* in Hipposideridae; *Nycteribia* sp. A (*n* = 23) on *M. pilosus* in Vespertilionidae; and *Nycteribia* sp. F (*n* = 7) on *M. pusillus* in Miniopteridae. *Nycteribia* sp. E also showed high host specificity (SI = 92.86, *n* = 13) to *M. pusillus* (Fig. 3 and Table 1).

Despite small sample sizes, two bat fly species were the only species associated with their host bat species, so these bat-bat fly relationships were also highly specific. They were nycteribiid *Basilia* sp. A (SI = 100, *n* = 2) and streblid *Brachytarsina* sp. C (SI = 100, *n* = 3), which were found on *M. horsfieldii* and *R. leschenaultii*, respectively (Fig. 3 and Table 1).

Streblid *Brachytarsina* spp. were detected on nine of the 11 bat species surveyed (except *M. horsfieldii* and *R. pusillus*). *Brachytarsina amboinensis* was prevalent (38% of total bat flies) and it showed the lowest host specificity (SI = 0.41–39.02) among all bat fly species, being found on six bat species in three families. It was abundant on *Miniopterus* but absent from *Rhinolophus* species. Other bat fly species with relatively low host specificity included *Brachytarsina* sp. B (SI = 3.84–61.54), which occurred on three bat species in two families such as *Rhinolophus* species, as well as nycteribiid *Penicillidia* sp. C (SI = 6.9–51.72), which was specific to *Miniopterus* but found on all three *Miniopterus* spp. (Fig. 3 and Table 1).

### Mitogenomes of three bat fly species

**Fig. 4:**
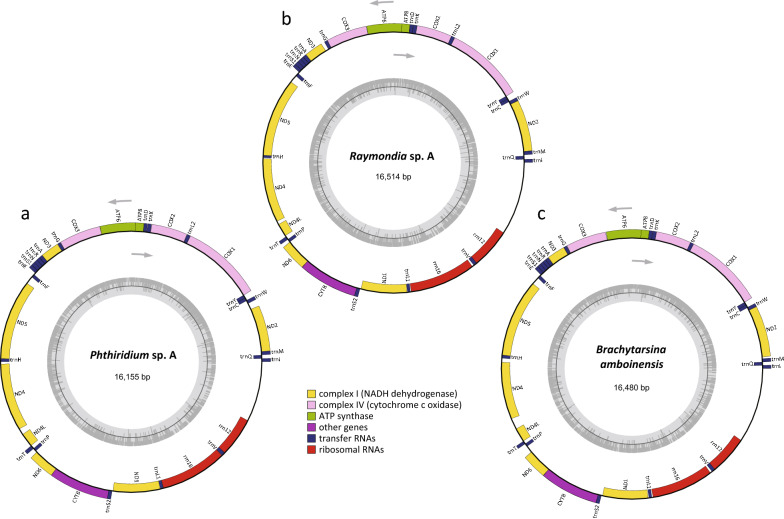
Mitogenomes of **a** *Phthiridium* sp. A, **b** *Raymondia* sp. A, and **c** *Brachytarsina amboinensis*. The genes labeled inside and outside of the cycle were encoded on the light and heavy strand, respectively. Genes in the same color code belong to the same gene family. The gray arrows indicate the direction of gene transcription. The grey circle in the middle shows the GC content of the mitogenome

The total number of mapped reads of *Phthiridium* sp. A, *B. amboinensis*, and *Raymondia* sp. A were 2029937, 891049, and 492479, respectively. The complete mitogenomes of *Phthiridium* sp. A, *B. amboinensis*, and *Raymondia* sp. A showed slight difference in sizes (Fig. 4). The total lengths of these three mitogenomes were 16155 bp, 16480 bp, and 16514 bp, respectively. These mitogenome sizes were very similar to those of other calyptrates (14–16 k bp), e.g. *Musca domestica* is 16108 bp, *Paratrichobius longicrus* is 16296 bp, and *Paradyschiria parvula* is 14588 bp [56, 57]. There are 37 genes within each mitogenome, including 13 protein-coding genes (PCGs), 22 transfer RNA genes (tRNAs), two ribosomal RNA genes (rRNAs), and one non-coding control region (CR) (Fig. 4). The organization and structures of genes in these three mitogenomes were identical, with 23 genes (including nine PCGs and 14 tRNAs) encoded on the heavy strand and 14 genes (including four PCGs, eight tRNAs and two rRNAs) encoded on the light strand.

## Discussion

Both evolutionary and ecological factors were suggested to be important for shaping the patterns of host association of bat flies, and some previous studies examined the significance of either one or both factors in explaining these patterns [58–60]. These studies demonstrated that bat flies generally showed variable degrees of host specialization and low cophylogenetic congruence to their hosts, indicating that extant patterns of bat-bat fly association may not be contributed by cospeciation. Remarkably, the bat-bat fly association unveiled in this study clearly showed that certain closely related bat flies within Nycteribiidae and Streblidae had affinities toward particular host bat species that are phylogenetically more closely related. For example, we observed that the congenerics of *Nycteribia* and conspecifics of *B. amboinensis* only infested hosts in *Myotis* and *Miniopterus*, which belong to two closely related families in Vespertilionoidea [61], whereas congenerics of *Raymondia* only parasitized hosts in *Rhinolophus* and *Hipposideros*, which are in two closely related families in Rhinolophoidea. These bat fly-bat genus associations observed were also supported by similar host associations of *Nycteribia* spp., *B. amboinensis*, and *Raymondia* spp. revealed in other nearby regions, such as Thailand and Malaysia [62, 63].

### From generalist to specialist: Bat-bat fly association

Elucidating the true distribution patterns of bat flies in a community of host bats is crucial for understanding the complex ecology of bat fly parasitism. With recent carefully controlled bat fly surveys, volant streblids in the Neotropics were reported as highly specialized [48, 64], with most species being monoxenous (i.e. infesting only a single host species). Less specialized species were also mainly associated with their primary host species [21, 65]. In this study, we found both generalist and specialist streblids that are monoxenous, stenoxenous (i.e. infesting two or more congeneric host species), or polyxenous (i.e. infesting two or more host genera) [65]. Recent records of *B. amboinensis* were mostly reported from the Philippines where the species infested multiple host species in *Miniopterus*, *Myotis*, *Rhinolophus*, and *Hipposideros* [66, 67]. However, according to older records, *B. amboinensis* also occurred in Taiwan and Japan, and those in Japan infested *M. fuliginosus* [68, 69]. Likewise, we found local *B. amboinensis* to be polyxenous, with specificity toward different host genera being unequal. About 77% *B. amboinensis* were associated with *M. magnater* and *M. pusillus*, whereas 22% parasitized *M. pilosus* and *M. chinensis*. Considering its flight capability, it was possible that the low host specificity was due to natural host switches or, alternatively but unlikely, random transfers [21]. Three other closely related *Brachytarsina* spp. were morphologically similar to *B. amboinensis*. However, unlike *B. amboinensis*, they appeared either monoxenous or stenoxenous. Over 96% of *Brachytarsina* sp. B were associated with *R. affinis* and *R. sinicus*, and *Brachytarsina* sp. D and sp. E were only found on *R. sinicus*. Thus, *B. amboinensis* did not share the same host species and specificity with other closely related *Brachytarsina* species. Notably, many individuals of other closely related *Brachytarsina* spp. and *B. amboinensis* were isolated from hosts captured in the same roosts on the same sampling dates, but only *B. amboinensis* showed this generalized association with several *Miniopterus* and *Myotis* species but not *Rhinolophus*. Thus, it is likely that *B. amboinensis* is a generalist parasite and exhibited natural dispersal between bat host species, predominantly *Miniopterus* and *Myotis*.

In addition, we found three streblid species in genus *Raymondia*. *Raymondia* sp. A was strictly monoxenous and only parasitized *H. gentilis*. Almost no other bat fly species and no nycteribiid were found on *H. gentilis*. The monoxeny of *Raymondia* sp. A could be in part attributed to their roosting behavior. *Hipposideros gentilis* often co-roost with other host species in caves, such as *R. pusillus*, but individuals of *H. gentilis* in colonies usually maintain certain interbat spacing and guard their area against intrusion [39], which might lessen inter-host switching of streblids. Moreover, the mobility of *Raymondia* spp. might be relatively limited by their tiny body sizes as *Raymondia* species were the smallest (< 1.5 mm) among all bat flies identified [31]. For *Raymondia* spp. B and C, we only secured one individual of each of these species, which infested *R. pusillus* and *R. affinis*, respectively. *Raymondia* sp. C is morphologically highly similar to *R. pagodarum*, which were found to mainly parasitize *Hipposideros* spp. and *Rhinolophus* spp. in Asian regions [70, 71]. *Hipposideros gentilis* was also one of these host species that *R. pagodarum* infested in Thailand [63]. *Raymondia pagodarum* and another species, *Raymondia molossia*, were also reported in China, but their hosts were unknown [31, 69].

In this study, we found more species in Nycteribiidae than Streblidae, which is consistent with the biogeographic pattern that Nycteribiidae species are primarily found in the Old World [10]. Compared to other identified nycteribiid genera, *Nycteribia* was the most species-rich group with five species found locally. *Nycteribia* sp. A was closely related to a *Nycteribia* sp. found in Hubei, China, whose primary host was unknown [72]. We found that *Nycteribia* sp. A was the only *Nycteribia* spp. we identified that parasitized local *Myotis* spp., and it was monoxenous and only infested *M. pilosus*. In Hong Kong, even though individuals of *M. pilosus* commonly mix with those of *Miniopterus* spp. and *M. chinensis* to form packed aggregations in roosts [39], and this roosting structure likely facilitates interspecific host exchanges of bat flies, *Nycteribia* sp. A was absent from other host species. *Nycteribia* spp. in proximate regions, including *Nycteribia quasiocellata* found in Manchuria in China, Mongolia, and Kazakhstan and a *Nycteribia* sp. in Thailand, were other congenerics that infested *Myotis petax* and *Myotis siligorensis*, respectively [63, 73].

In the phylogenetic tree, two divergent clades in *Nycteribia* contain the GenBank sequences identified as *N. allotopa*; thus, some *N. allotopa* in the tree were likely misidentified. *Nycteribia* sp. B was identical to a *Nycteribia* species claimed to be *N. allotopa* in Wakayama, Japan, associated with *M. fuliginosus* [74]. *Nycteribia allotopa* was also reported in Taiwan, Korea, and Thailand where they also lived on *M. fuliginosus* [63, 68, 75, 76]. In Hong Kong, there are three sympatric *Miniopterus* species, including *M. magnater*, *M. pusillus*, and *M. fuliginosus*, which are morphologically similar. Individuals of *M. magnater* or *M. fuliginosus* occasionally form tightly packed and mixed assemblages with those of *M. pusillus*, but co-roosting of *M. magnater* and *M. fuliginosus* is locally rare (AFCD unpublished data). Our results indicated that *Nycteribia* sp. B was stenoxenous and parasitized all three *Miniopterus* species; it was the only *Nycteribia* found on *M. fuliginosus*. Notably, the distribution ranges of *M. magnater* and *M. pusillus* do not include Japan, Korea, and Taiwan [77]. Moreover, *N. allotopa* was also reported to be found on other host species that were absent from Hong Kong, such as other species in *Miniopterus*, *Rhinolophus*, *Pipistrellus*, *Megaderma*, and *Tadarida* in nearby regions [71, 78–80]. These records might reflect the potential of *Nycteribia* sp. B in inter-host switching. The other two *Nycteribia* spp., *Nycteribia* sp. D and sp. E, were closely related to the *Nycteribia* that described as *N. allotopa* in Wakayama, Japan [81]. Yet unlike *N. allotopa*, *Nycteribia* sp. D and sp. E were not found on *M. fuliginosus* but predominantly infested *M. magnater* and *M. pusillus*, respectively.

Another identified nycteribiid genus in which the congenerics were exclusively associated with *Miniopterus* was *Penicillidia*. We found that *Penicillidia* spp. A, B, and C were all stenoxenous. *Penicillidia* sp. A was closely related to *P. oceanica* in the Philippines which infested *Miniopterus schreibersi* [82]. It parasitized both *M. magnater* and *M. fuliginosus* that rarely co-roost in Hong Kong. *Penicillidia* sp. B was associated with *M. magnater* and *M. pusillus*, which often co-roost. *Penicillidia* sp. C were also found on *M. fuliginosus*. Other congeneric species, e.g. *Penicillidia jenynsii* in Japan, also infested *Miniopterus* species [68]. *Penicillidia monoceros* was reported to occur in Hubei in China and Mongolia, but those in Mongolia were reported to primarily infest *Myotis petax* [72, 73]. Notably, we observed that *Miniopterus* spp. hosted the most diverse communities of bat fly species, with the number of species ranging from four to seven. Contrary to other bat genera, the bat fly species assemblages among the three congenerics of *Miniopterus* largely overlapped with each other, and they shared at least three bat fly species, including *B. amboinensis*, *Nycteribia* sp. B, and *Penicillidia* sp. C. Due in part to the gregarious habits of *Miniopterus* species and their propensity to form tightly packed clusters in caves, *Miniopterus* individuals offered various opportunities to horizontally transfer their bat flies, and these bat flies could find mates readily on multiple host species to reproduce, so adapting and exploiting tightly packed hosts in multiple congenerics should increase the abundance and overall fitness of these bat flies [18]. Slight intra-specific morphological variations were also observed within populations of *Penicillidia* spp. B and C; it was proposed that phenotypic plasticity of an ectoparasitic species might confer greater fitness advantage in their ability to parasitize a wider range of host species [58].

While *Penicillidia* was found on *Miniopterus* exclusively, identified species in *Phthiridium* were exclusively associated with *Rhinolophus*. *Phthiridium* sp. A was a close relative to *P. hindlei* found in Osaka, Japan, that lived on *Rhinolophus ferrumequinum* [81]. Records of other examples of *Phthiridium* spp. in China included *P. hindlei* infesting *R. ferrumequinum* in Shandong, *P. szechuanum* found on *R. pusillus* in Sichuan, and *P. ornatum* parasitizing *Rhinolophus* sp. in Yunnan [25, 69, 83]. Although *Phthiridium* sp. A was very abundant on *R. sinicus* and individuals of *R. sinicus* can form packed and mixed assemblages with those of *R. affinis* in Hong Kong, especially during winter, *Phthiridium* sp. A was strictly monoxenous. It was also the only nycteribiid species found on *R. sinicus*, even though *R. sinicus* co-roosts with *M. pusillus* and *M. pilosus*, for example, which harbor other nycteribiids. Dispersal limitations, adaptative limitations, and reproductive isolation were several main explanations previously proposed to broadly account for how high host specificity of bat flies might evolve and be maintained [18]. However, mechanisms underlying bat-bat fly relationships are complex, and most research on our studied bat fly genera mainly focused on host-parasite occurrences and interactions; studies dealing with the ecology and biology of these studied genera and species in detail were scarce. It remains of interest to investigate which factors are involved and how they might control the host specificity of these bat fly species.

### Challenges and future directions

On a final note, we would like to highlight the challenge of species identification based on old taxonomic keys [31, 33, 68, 70], especially for regions with limited prior information on the bat fly community. Moreover, cryptic species and morphological plasticity are likely to be common in bat flies [29, 84, 85], making existing taxonomic keys insufficient for accurate species identification. In this study, most bat flies were morphologically distinct except a few cryptic species that were challenging to differentiate. For example, *Brachytarsina* spp. B, D, and E were morphologically highly similar and shared the same hosts, but they were differentiated by genetic divergence. Slight variations in morphology were observed among conspecifics of *Penicillidia* spp. B and C, respectively, but revealed to be intraspecific variations by DNA barcoding results. Therefore, as suggested by some recent studies [13, 37], we advocate identifying bat fly species using morphological characteristics with the support of DNA barcoding to discern bat-bat fly associations accurately in future studies. In addition to genetic data, provision of high-quality photos and formal species description with the development of new taxonomic keys will be crucial resources for efficient bat fly classification, which are especially important for understudied regions potentially with many undescribed species.

## Conclusions

In this study, we found 20 bat fly species from a subtropical region in East Asia in which many of them appear to be new records. We have also unveiled the associations of these bat fly species to host bat species and revealed a range of host specificity among these bat flies. However, detailed information on the biology and ecology of bat flies, as well as host bat species, occurring in the East Asia region remains limited, which makes elucidation of the complex mechanisms underlying bat-bat fly associations difficult. More studies on bat flies and bats in the region, such as from bat fly species discovery to their distribution and behaviors on hosts, will be essential to better understand how various evolutionary or ecological factors shape the extant bat-bat fly relationships in the regions.

## Supplementary Information

**Additional file 1: Figure S1.** Images of *Nycteribia* sp. A: (a) dorsal and (b) ventral views of male and (c) dorsal and (d) ventral views of female. Scale bar = 1 mm. **Figure S2.** Images of *Nycteribia* sp. B: (a) dorsal and (b) ventral views of male and (c) dorsal and (d) ventral views of female. Scale bar = 1 mm. **Figure S3.** Images of *Nycteribia* sp. D: (a) dorsal and (b) ventral views of male and (c) ventral and (d) dorsal views of female. Scale bar = 1 mm. **Figure S4.** Images of *Nycteribia* sp. E: (a) dorsal and (b) ventral views of male and (c) ventral and (d) dorsal views of female. Scale bar = 1 mm. **Figure S5.** Images of *Nycteribia* sp. F: (a) dorsal and (b) ventral views of male and (c) dorsal and (d) ventral views of female. Scale bar = 1 mm. **Figure S6.** Images of *Phthiridium* sp. A: (a) dorsal and (b) ventral views of male and (c) dorsal and (d) ventral views of female. Scale bar = 1 mm. **Figure S7.** Images of *Phthiridium* sp. B: (a) dorsal and (b) ventral views, male only. Scale bar = 1 mm. **Figure S8.** Images *Basilia* sp. A: (a) dorsal and (b) ventral views, female only. Scale bar = 1 mm. **Figure S9.** Images of *Penicillidia* sp. A: (a) ventral and (b) dorsal views of male and (c) ventral and (d) dorsal views of female. Scale bar = 1 mm. **Figure S10.** Images of *Penicillidia* sp. B: (a) dorsal and (b) ventral views of male and (c) dorsal and (d) ventral views of female. Scale bar = 1 mm. **Figure S11.** Images of *Penicillidia* sp. C: (a) ventral and (b) dorsal views of male and (c) dorsal and (d) ventral views of female. Scale bar = 1 mm. **Figure S12.** Images of *Nycteribiidae* species A: (a) dorsal and (b) ventral views, male only, scale bar unavailable. **Figure S13.** Images of *Raymondia* sp. A: (a) dorsal and (b) ventral views of female and (c) dorsal and (d) ventral views of male. Scale bar = 1 mm. **Figure S14.** Images of *Raymondia* sp. B: (a) dorsal and (b) ventral views, male only, scale bar unavailable. **Figure S15.** Images of *Raymondia* sp. C: (a) and (c) ventral and (b) dorsal views, female only. Scale bar = 1 mm for (a) and (b) and no scale bar available for (c). Images of (a) and (b) shown were captured from a protease-digested specimen and abdomen was contracted. **Figure S16.** Images of *Brachytarsina amboinensis*: (a) dorsal and (b) ventral views of male and (c) dorsal and (d) ventral views of female. Scale bar = 1 mm. **Figure S17.** Images of *Brachytarsina* sp. B: (a) dorsal and (b) ventral views of male and (c) dorsal and (d) ventral views of female. Scale bar = 1 mm. **Figure S18.** Images of *Brachytarsina* sp. C: (a) dorsal and (b) ventral views of female, scale bar = 1 mm; (c) ventral and (d) dorsal views of male, scale bar unavailable. **Figure S19.** Images of *Brachytarsina* sp. D: (a) dorsal and (b) ventral views of male and (c) dorsal and (d) ventral views of female. Images of the female shown were captured from a protease-digested specimen. Scale bar = 1 mm. **Figure S20.** Images of *Brachytarsina* sp. E: (a) dorsal and (b) ventral views, male only. Scale bar = 1 mm. **Figure S21.** Phylogenetic relationships of all DNA barcoded individuals of the 20 bat fly species identified in this study, inferred from the (a) Bayesian inference (BI) and (b) maximum likelihood (ML) methods based on the COI gene (609 bp). The values before each node represent the Bayesian posterior probability and the ML bootstrap value.

## Data Availability

The GenBank accession numbers of the bat fly DNA sequences and mitogenomes are OQ184520–184639 and OQ301747–301749, respectively.
