# Supplementary material for: Species richness of bat flies and their associations with host bats in a subtropical East Asian region
Source: Parasit Vectors. 2023 Jan 27;16:37. doi: 10.1186/s13071-023-05663-x (PMC9881358; doi:10.1186/s13071-023-05663-x)
Supplement: Supplementary file 1 — Additional file 1: Figure S1. Images of Nycteribia sp. A: (a) dorsal and (b) ventral views of male and (c) dorsal and (d) ventral views of female. Scale bar = 1 mm. Figure S2. Images of Nycteribia sp. B: (a) dorsal and (b) ventral views of male and (c) dorsal and (d) ventral views of female. Scale bar = 1 mm. Figure S3. Images of Nycteribia sp. D: (a) dorsal and (b) ventral views of male and (c) ventral and (d) dorsal views of female. Scale bar = 1 mm. Figure S4. Images of Nycteribia sp. E: (a) dorsal and (b) ventral views of male and (c) ventral and (d) dorsal views of female. Scale bar = 1 mm. Figure S5. Images of Nycteribia sp. F: (a) dorsal and (b) ventral views of male and (c) dorsal and (d) ventral views of female. Scale bar = 1 mm. Figure S6. Images of Phthiridium sp. A: (a) dorsal and (b) ventral views of male and (c) dorsal and (d) ventral views of female. Scale bar = 1 mm. Figure S7. Images of Phthiridium sp. B: (a) dorsal and (b) ventral views, male only. Scale bar = 1 mm. Figure S8. Images Basilia sp. A: (a) dorsal and (b) ventral views, female only. Scale bar = 1 mm. Figure S9. Images of Penicillidia sp. A: (a) ventral and (b) dorsal views of male and (c) ventral and (d) dorsal views of female. Scale bar = 1 mm. Figure S10. Images of Penicillidia sp. B: (a) dorsal and (b) ventral views of male and (c) dorsal and (d) ventral views of female. Scale bar = 1 mm. Figure S11. Images of Penicillidia sp. C: (a) ventral and (b) dorsal views of male and (c) dorsal and (d) ventral views of female. Scale bar = 1 mm. Figure S12. Images of Nycteribiidae species A: (a) dorsal and (b) ventral views, male only, scale bar unavailable. Figure S13. Images of Raymondia sp. A: (a) dorsal and (b) ventral views of female and (c) dorsal and (d) ventral views of male. Scale bar = 1 mm. Figure S14. Images of Raymondia sp. B: (a) dorsal and (b) ventral views, male only, scale bar unavailable. Figure S15. Images of Raymondia sp. C: (a) and (c) ventral and (b) dorsal views, female o [file 13071_2023_5663_MOESM1_ESM.pdf]

Supplementary Materials for

Species richness of bat flies and their associations with host bats in a subtropical East Asian region

Figures S1- S21

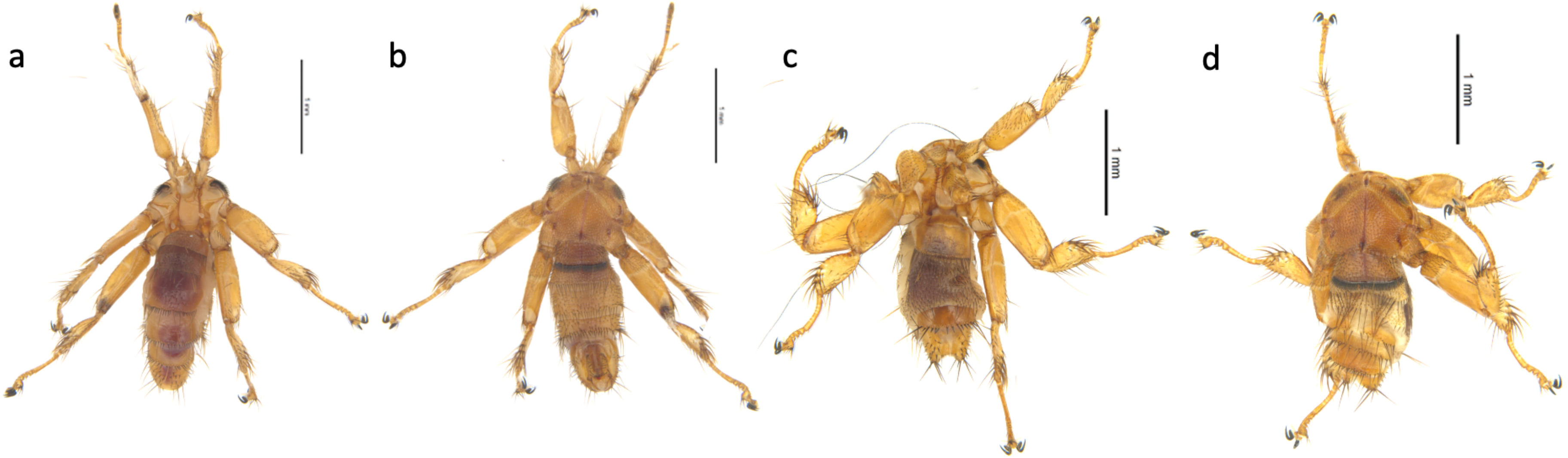

Figure S1 Images of *Nycteribia* sp. A, a) dorsal and b) ventral views of male, and c) dorsal and d) ventral views of female. Scale bar = 1mm.

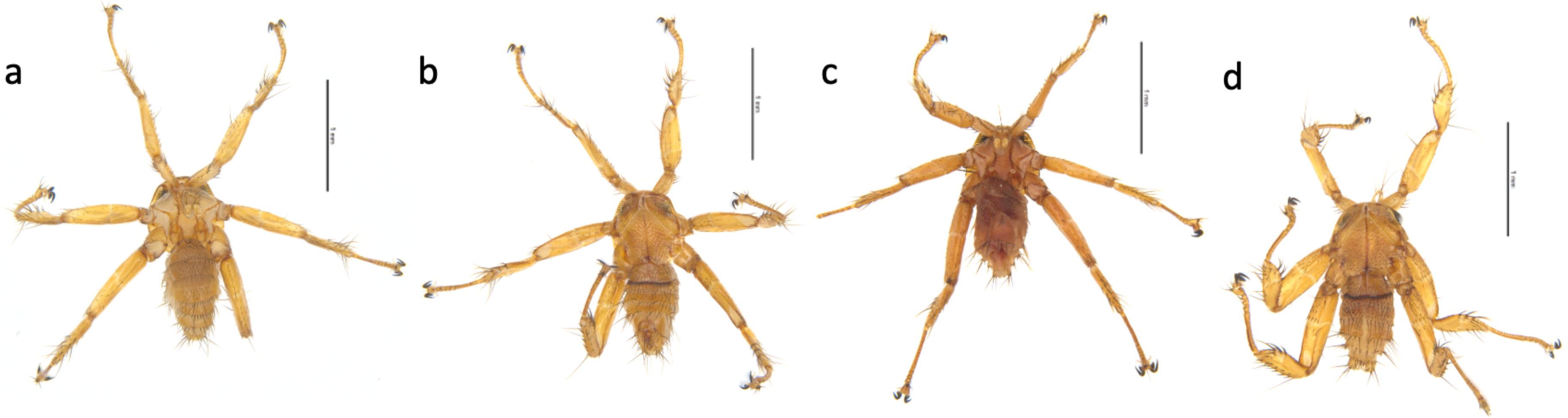

Figure S2 Images of *Nycteribia* sp. B, a) dorsal and b) ventral views of male, and c) dorsal and d) ventral views of female. Scale bar = 1mm.

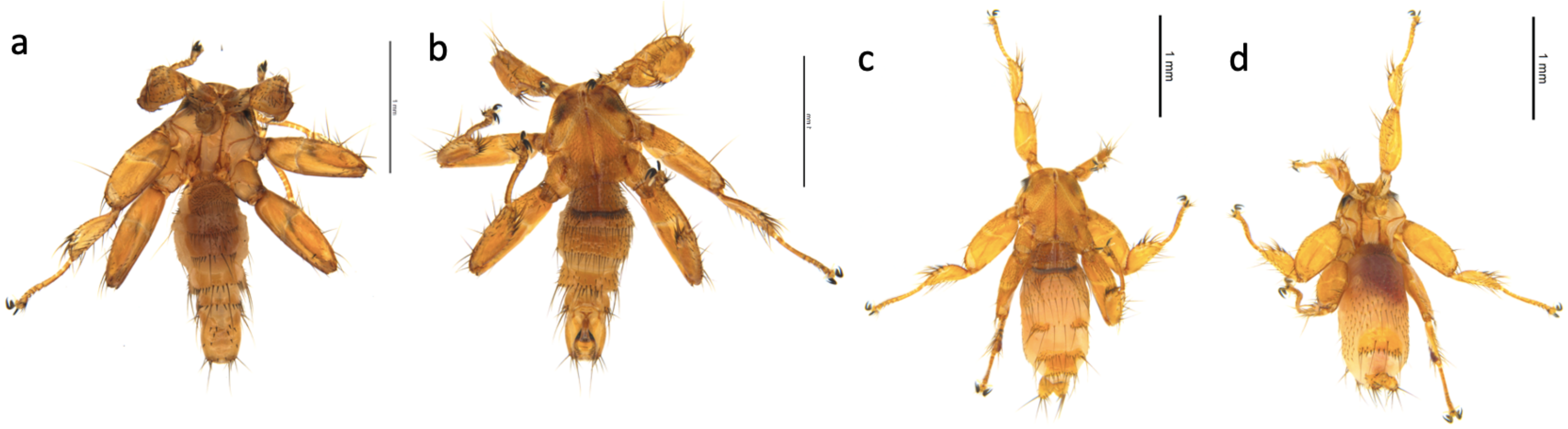

Figure S3 Images of *Nycteribia* sp. D, a) dorsal and b) ventral views of male, and c) ventral and d) dorsal views of female. Scale bar = 1mm.

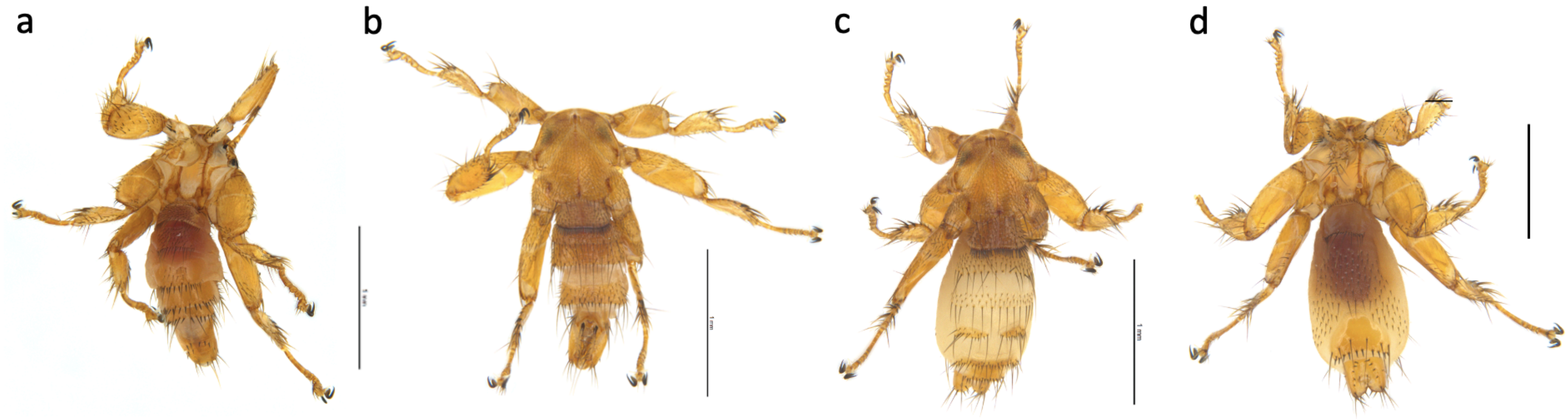

Figure S4 Images of *Nycteribia* sp. E, a) dorsal and b) ventral views of male, and c) ventral and d) dorsal views of female. Scale bar = 1mm.

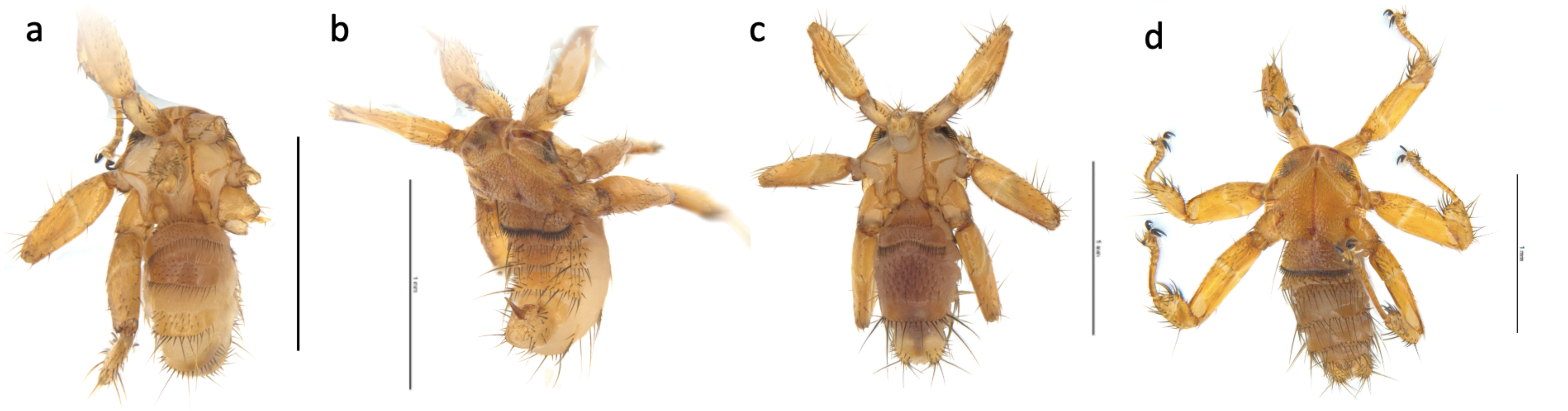

Figure S5 Images of *Nycteribia* sp. F, a) dorsal and b) ventral views of male, and c) dorsal and d) ventral views of female. Scale bar = 1mm.

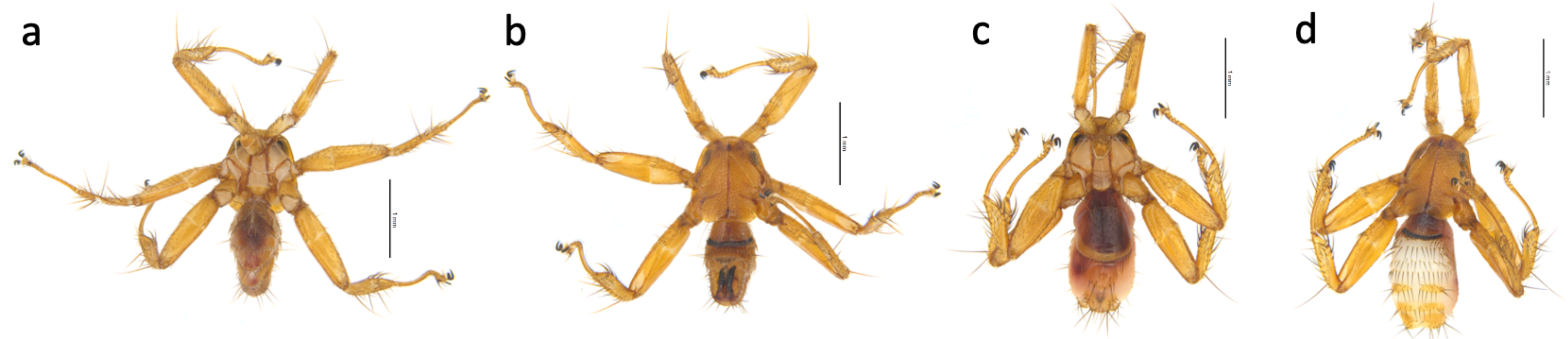

Figure S6 Images of *Phthiridium* sp. A, a) dorsal and b) ventral views of male, and c) dorsal and d) ventral views of female. Scale bar = 1mm.

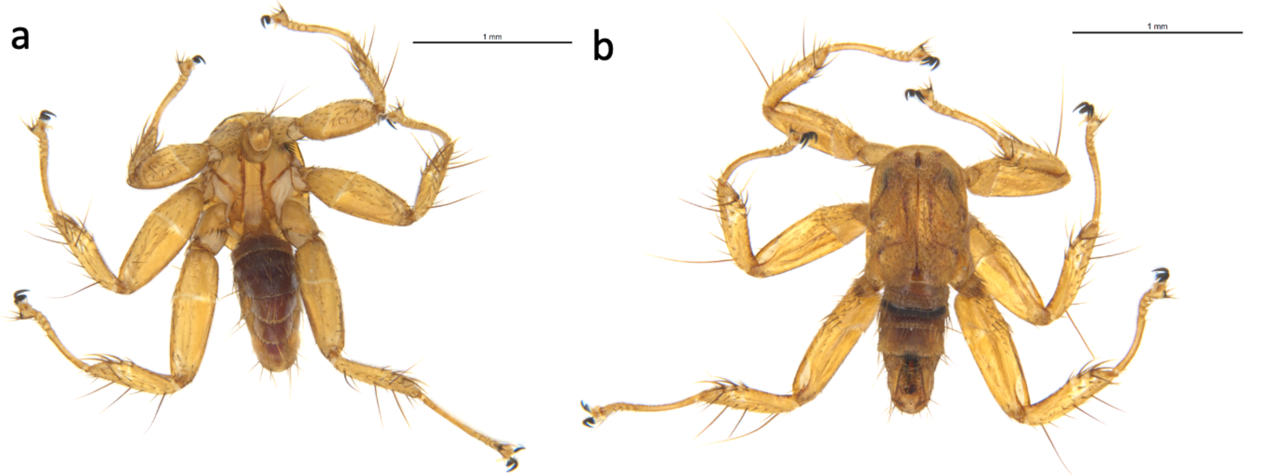

Figure S7 Images of *Phthiridium* sp. B, a) dorsal and b) ventral views, male only. Scale bar = 1mm.

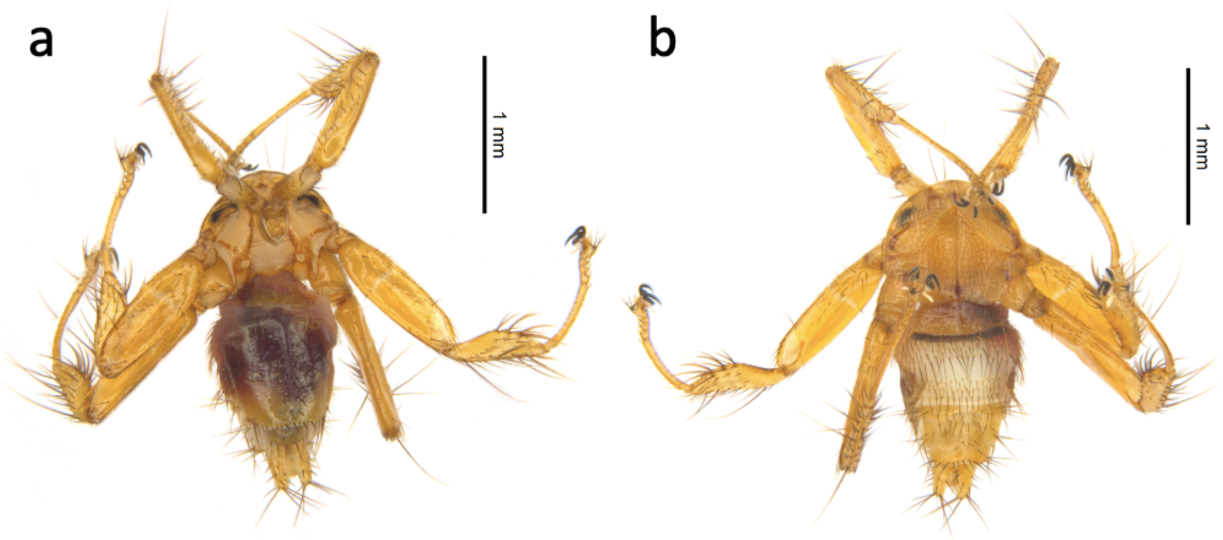

Figure S8 Images *Basilia* sp. A, a) dorsal and b) ventral views, female only. Scale bar = 1mm.

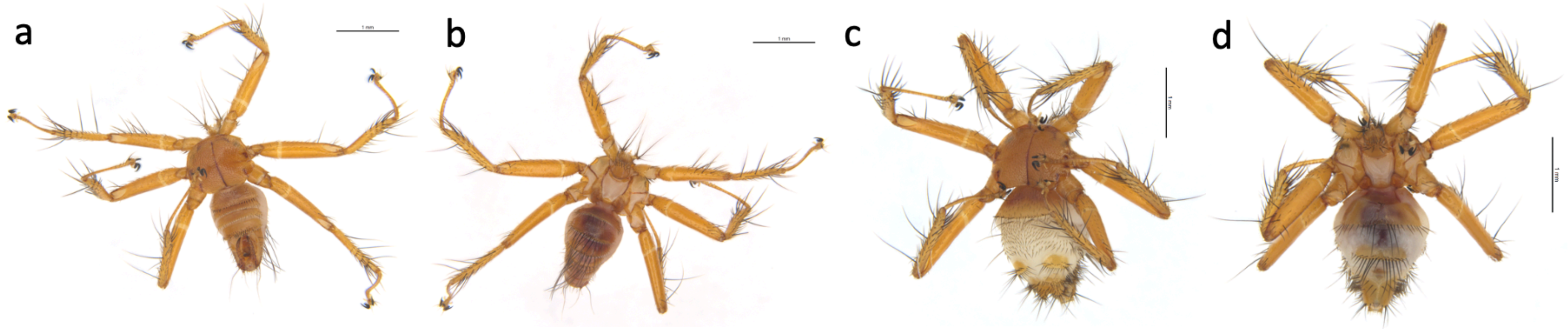

Figure S9 Images of *Penicillidia* sp. A, a) ventral and b) dorsal views of male, and c) ventral and d) dorsal views of female. Scale bar = 1mm.

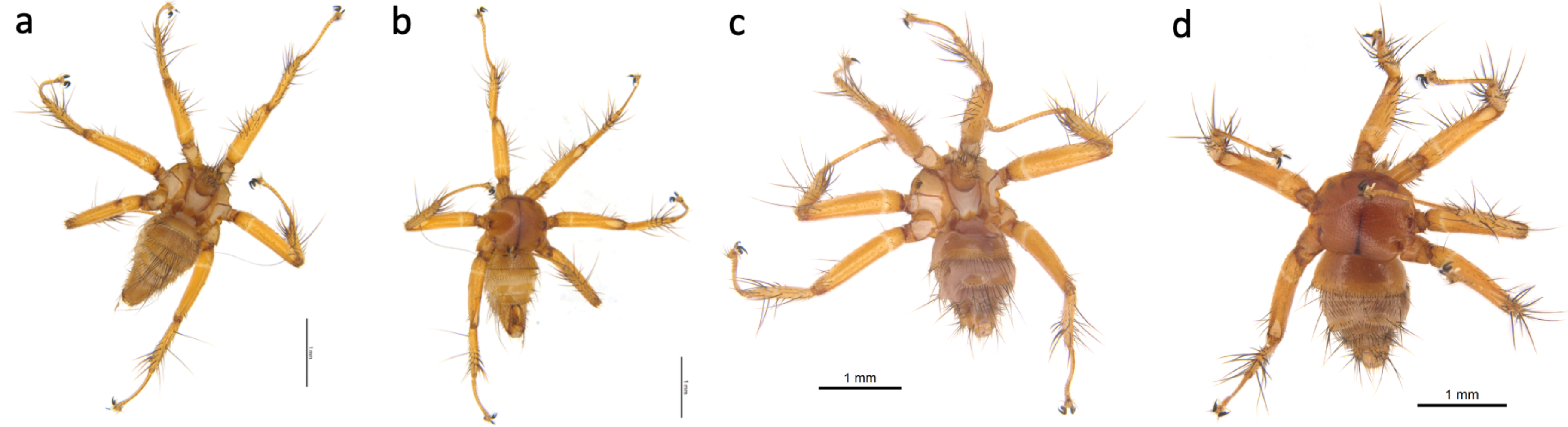

Figure S10 Images of *Penicillidia* sp. B, a) dorsal and b) ventral views of male, and c) dorsal and d) ventral views of female. Scale bar = 1mm.

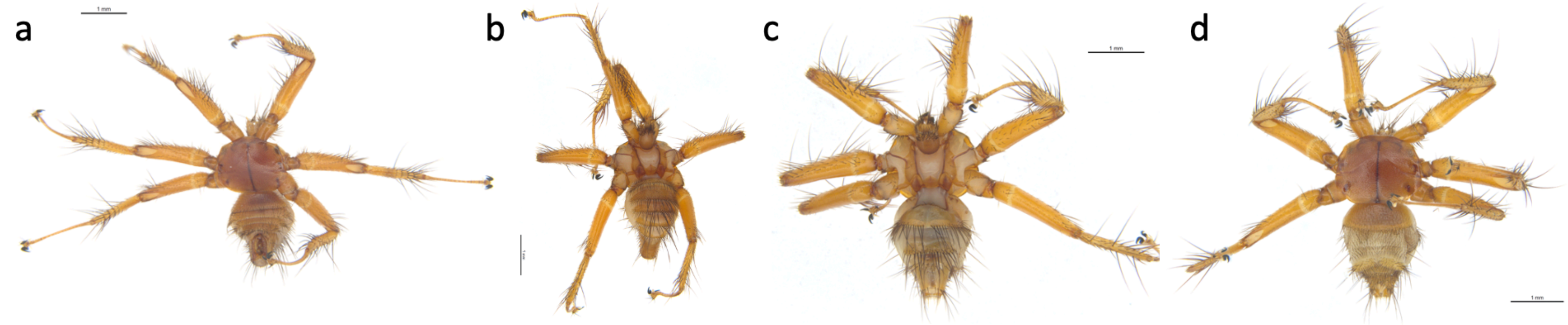

Figure S11 Images of *Penicillidia* sp. C, a) ventral and b) dorsal views of male, and c) dorsal and d) ventral views of female. Scale bar = 1mm.

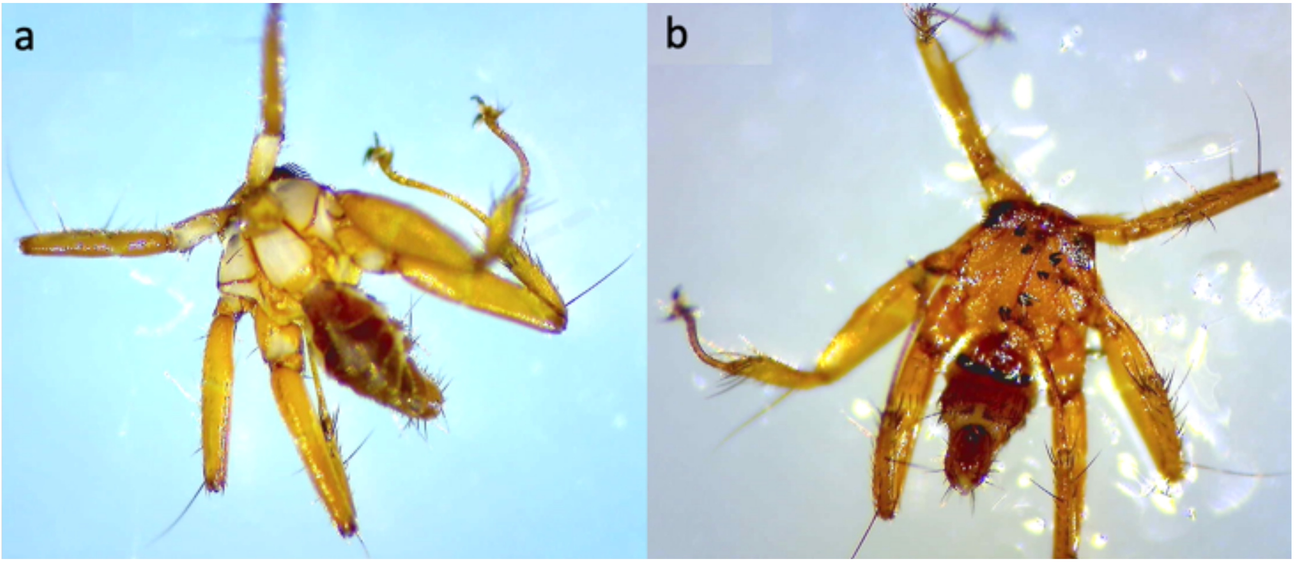

Figure S12 Images of Nycteribiidae species A, a) dorsal and b) ventral views, male only, scale bar unavailable.

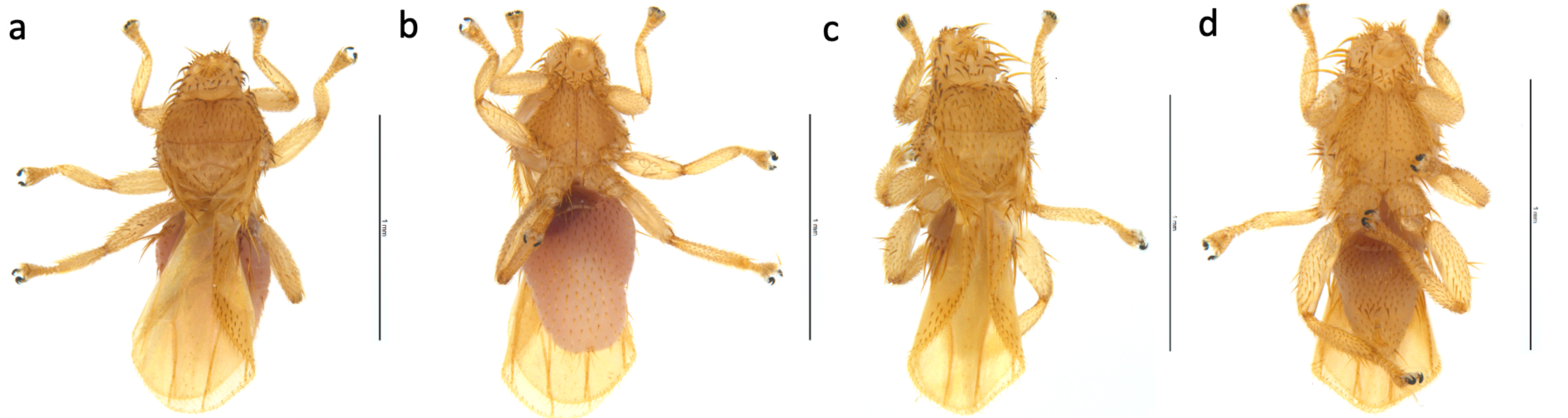

Figure S13 Images of *Raymondia* sp. A, a) dorsal and b) ventral views of female, and c) dorsal and d) ventral views of male. Scale bar = 1mm.

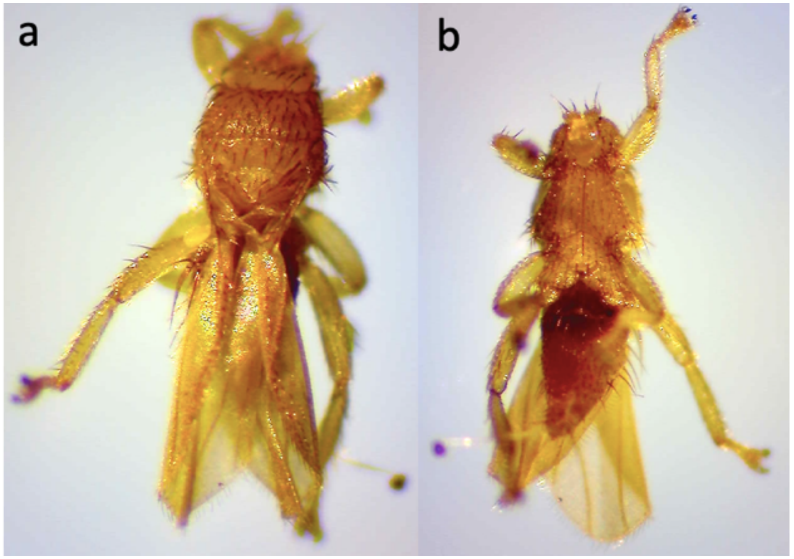

Figure S14 Images of *Raymondia* sp. B, a) dorsal and b) ventral views, male only, scale bar unavailable.

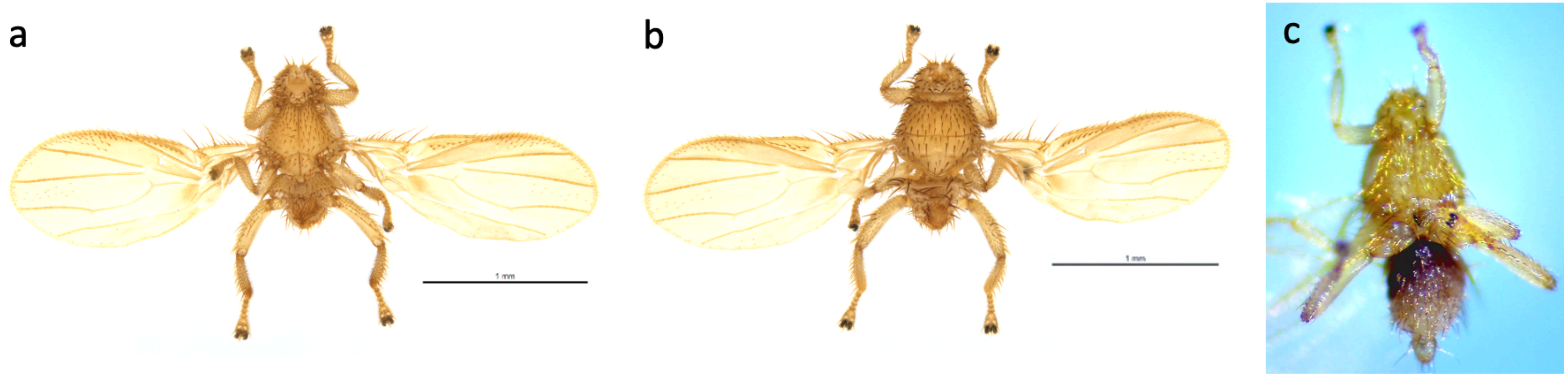

Figure S15 Images of *Raymondia* sp. C, a) and c) ventral and b) dorsal views, female only. Scale bar = 1mm for a) and b) and no scale bar available for c). Images of a) and b) shown were captured from a protease-digested specimen and abdomen was contracted.

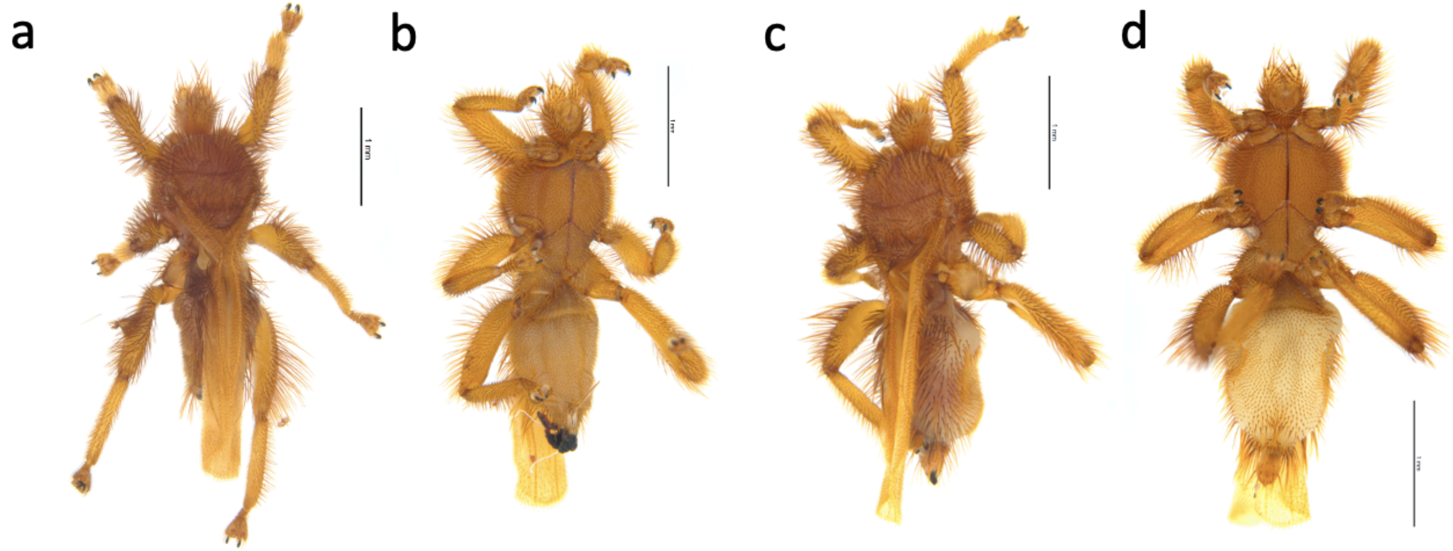

Figure S16 Images of *Brachytarsina amboinensis*, a) dorsal and b) ventral views of male, and c) dorsal and d) ventral views of female. Scale bar = 1mm.

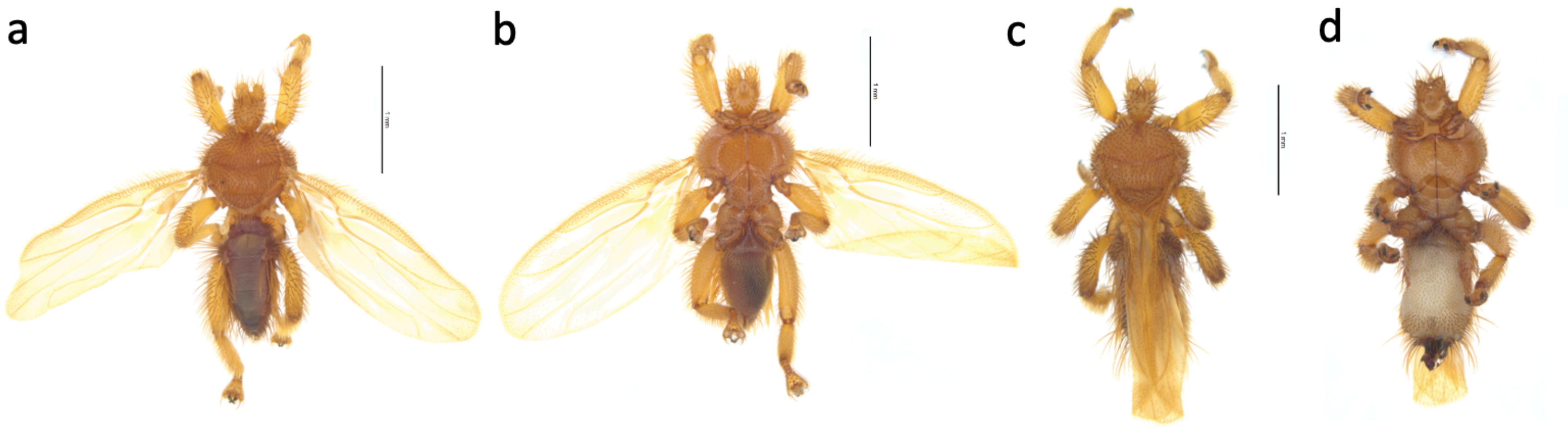

Figure S17 Images of *Brachytarsina* sp. B, a) dorsal and b) ventral views of male, and c) dorsal and d) ventral views of female. Scale bar = 1mm.

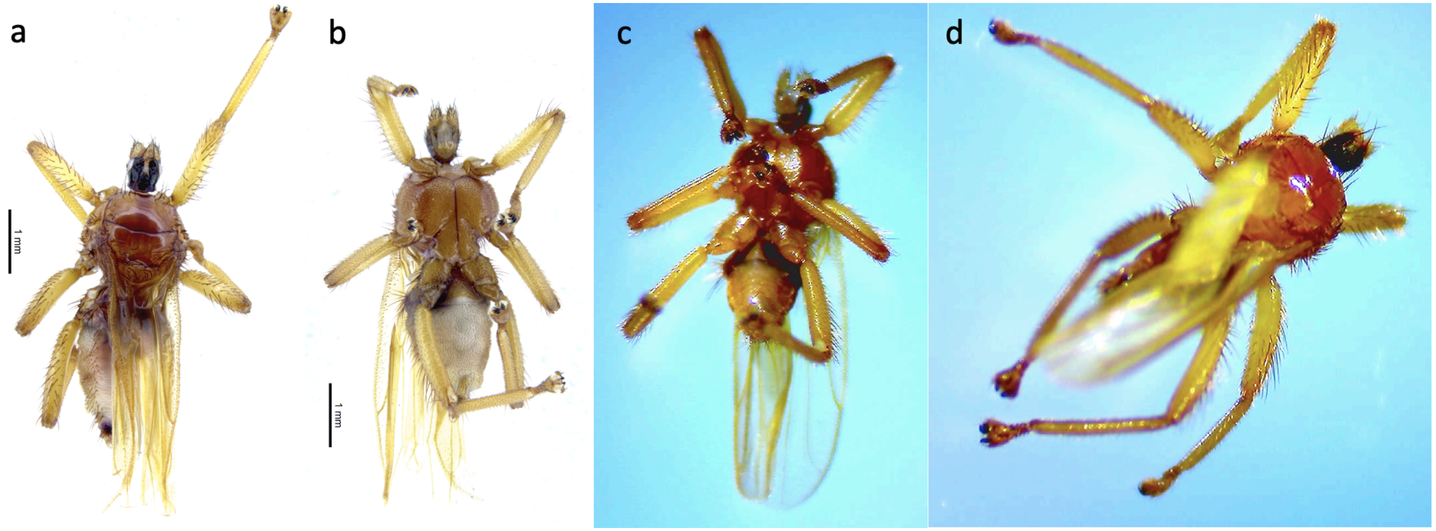

Figure S18 Images of *Brachytarsina* sp. C, a) dorsal and b) ventral views of female, scale bar = 1mm; c) ventral and d) dorsal views of male, scale bar unavailable.

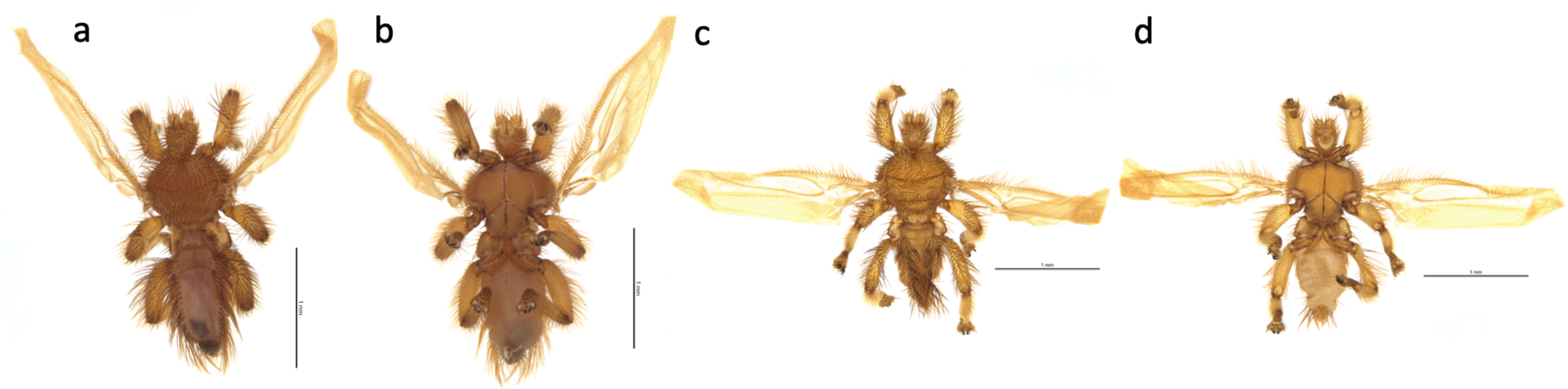

Figure S19 Images of *Brachytarsina* sp. D, a) dorsal and b) ventral views of male, and c) dorsal and d) ventral views of female. Images of the female shown were captured from a protease-digested specimen. Scale bar = 1mm.

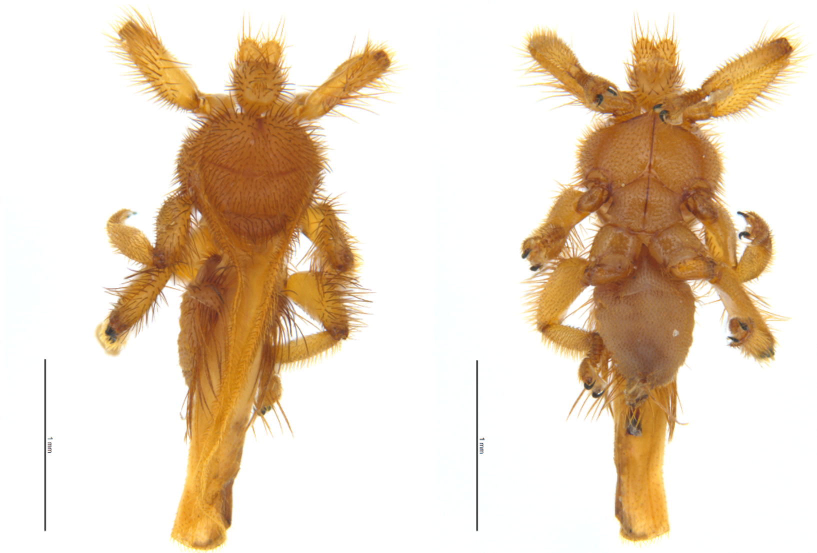

Figure S20 Images of *Brachytarsina* sp. E, a) dorsal and b) ventral views, male only. Scale bar = 1mm.

a)

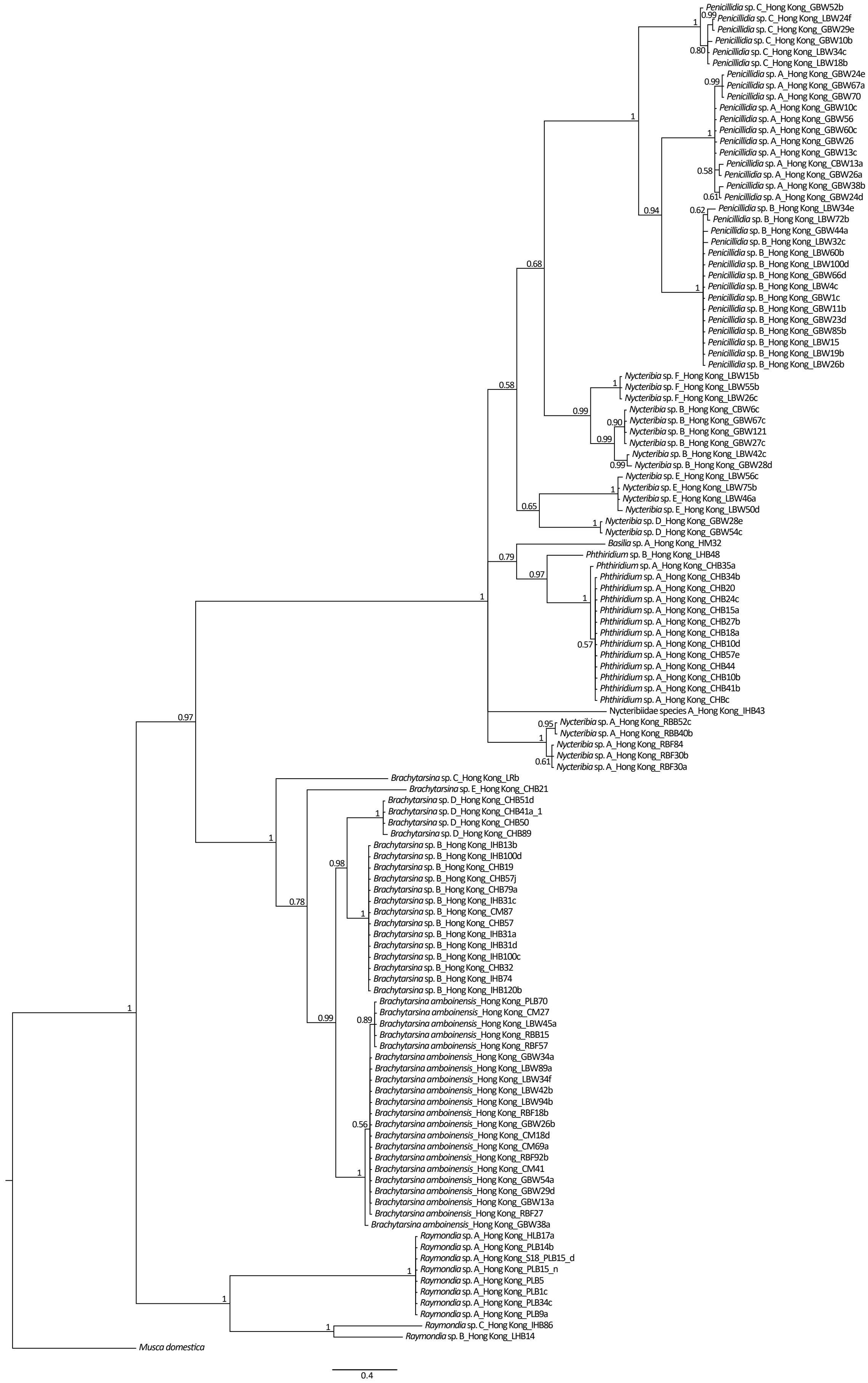
Penicillidia sp. C_Hong Kong_GBW52b
Penicillidia sp. C_Hong Kong_LBW24f
Penicillidia sp. C_Hong Kong_GBW29e
Penicillidia sp. C_Hong Kong_GBW10b
Penicillidia sp. C_Hong Kong_LBW34c
Penicillidia sp. C_Hong Kong_LBW18b
Penicillidia sp. A_Hong Kong_GBW24e
Penicillidia sp. A_Hong Kong_GBW67a
Penicillidia sp. A_Hong Kong_GBW70
Penicillidia sp. A_Hong Kong_GBW10c
Penicillidia sp. A_Hong Kong_GBW56
Penicillidia sp. A_Hong Kong_GBW60c
Penicillidia sp. A_Hong Kong_GBW26
Penicillidia sp. A_Hong Kong_GBW13c
Penicillidia sp. A_Hong Kong_CBW13a
Penicillidia sp. A_Hong Kong_GBW26a
Penicillidia sp. A_Hong Kong_GBW38b
Penicillidia sp. A_Hong Kong_GBW24d
Penicillidia sp. B_Hong Kong_LBW34e
Penicillidia sp. B_Hong Kong_LBW72b
Penicillidia sp. B_Hong Kong_GBW44a
Penicillidia sp. B_Hong Kong_LBW32c
Penicillidia sp. B_Hong Kong_LBW60b
Penicillidia sp. B_Hong Kong_LBW100d
Penicillidia sp. B_Hong Kong_GBW66d
Penicillidia sp. B_Hong Kong_LBW4c
Penicillidia sp. B_Hong Kong_GBW1c
Penicillidia sp. B_Hong Kong_GBW11b
Penicillidia sp. B_Hong Kong_GBW23d
Penicillidia sp. B_Hong Kong_GBW85b
Penicillidia sp. B_Hong Kong_LBW15
Penicillidia sp. B_Hong Kong_LBW19b
Penicillidia sp. B_Hong Kong_LBW26b
Nycteribia sp. F_Hong Kong_LBW15b
Nycteribia sp. F_Hong Kong_LBW55b
Nycteribia sp. F_Hong Kong_LBW26c
Nycteribia sp. B_Hong Kong_CBW6c
Nycteribia sp. B_Hong Kong_GBW67c
Nycteribia sp. B_Hong Kong_GBW121
Nycteribia sp. B_Hong Kong_GBW27c
Nycteribia sp. B_Hong Kong_LBW42c
Nycteribia sp. B_Hong Kong_GBW28d
Nycteribia sp. E_Hong Kong_LBW56c
Nycteribia sp. E_Hong Kong_LBW75b
Nycteribia sp. E_Hong Kong_LBW46a
Nycteribia sp. E_Hong Kong_LBW50d
Nycteribia sp. D_Hong Kong_GBW28e
Nycteribia sp. D_Hong Kong_GBW54c
Basilia sp. A_Hong Kong_HM32
Phthiridium sp. B_Hong Kong_LHB48
Phthiridium sp. A_Hong Kong_CHB35a
Phthiridium sp. A_Hong Kong_CHB34b
Phthiridium sp. A_Hong Kong_CHB20
Phthiridium sp. A_Hong Kong_CHB24c
Phthiridium sp. A_Hong Kong_CHB15a
Phthiridium sp. A_Hong Kong_CHB27b
Phthiridium sp. A_Hong Kong_CHB18a
Phthiridium sp. A_Hong Kong_CHB10d
Phthiridium sp. A_Hong Kong_CHB57e
Phthiridium sp. A_Hong Kong_CHB44
Phthiridium sp. A_Hong Kong_CHB10b
Phthiridium sp. A_Hong Kong_CHB41b
Phthiridium sp. A_Hong Kong_CHBc
Nycteribiidae species A_Hong Kong_IHB43
Nycteribia sp. A_Hong Kong_RBB52c
Nycteribia sp. A_Hong Kong_RBB40b
Nycteribia sp. A_Hong Kong_RBF84
Nycteribia sp. A_Hong Kong_RBF30b
Nycteribia sp. A_Hong Kong_RBF30a
Brachytarsina sp. C_Hong Kong_LRb
Brachytarsina sp. E_Hong Kong_CHB21
Brachytarsina sp. D_Hong Kong_CHB51d
Brachytarsina sp. D_Hong Kong_CHB41a_1
Brachytarsina sp. D_Hong Kong_CHB50
Brachytarsina sp. D_Hong Kong_CHB89
Brachytarsina sp. B_Hong Kong_IHB13b
Brachytarsina sp. B_Hong Kong_IHB100d
Brachytarsina sp. B_Hong Kong_CHB19
Brachytarsina sp. B_Hong Kong_CHB57j
Brachytarsina sp. B_Hong Kong_CHB79a
Brachytarsina sp. B_Hong Kong_IHB31c
Brachytarsina sp. B_Hong Kong_CM87
Brachytarsina sp. B_Hong Kong_CHB57
Brachytarsina sp. B_Hong Kong_IHB31a
Brachytarsina sp. B_Hong Kong_IHB31d
Brachytarsina sp. B_Hong Kong_IHB100c
Brachytarsina sp. B_Hong Kong_CHB32
Brachytarsina sp. B_Hong Kong_IHB74
Brachytarsina sp. B_Hong Kong_IHB120b
Brachytarsina amboinensis_Hong Kong_PLB70
Brachytarsina amboinensis_Hong Kong_CM27
Brachytarsina amboinensis_Hong Kong_LBW45a
Brachytarsina amboinensis_Hong Kong_RBB15
Brachytarsina amboinensis_Hong Kong_RBF57
Brachytarsina amboinensis_Hong Kong_GBW34a
Brachytarsina amboinensis_Hong Kong_LBW89a
Brachytarsina amboinensis_Hong Kong_LBW34f
Brachytarsina amboinensis_Hong Kong_LBW42b
Brachytarsina amboinensis_Hong Kong_LBW94b
Brachytarsina amboinensis_Hong Kong_RBF18b
Brachytarsina amboinensis_Hong Kong_GBW26b
Brachytarsina amboinensis_Hong Kong_CM18d
Brachytarsina amboinensis_Hong Kong_CM69a
Brachytarsina amboinensis_Hong Kong_RBF92b
Brachytarsina amboinensis_Hong Kong_CM41
Brachytarsina amboinensis_Hong Kong_GBW54a
Brachytarsina amboinensis_Hong Kong_GBW29d
Brachytarsina amboinensis_Hong Kong_GBW13a
Brachytarsina amboinensis_Hong Kong_RBF27
Brachytarsina amboinensis_Hong Kong_GBW38a
Raymondia sp. A_Hong Kong_HLB17a
Raymondia sp. A_Hong Kong_PLB14b
Raymondia sp. A_Hong Kong_S18_PLB15_d
Raymondia sp. A_Hong Kong_PLB15_n
Raymondia sp. A_Hong Kong_PLB5
Raymondia sp. A_Hong Kong_PLB1c
Raymondia sp. A_Hong Kong_PLB34c
Raymondia sp. A_Hong Kong_PLB9a
Raymondia sp. C_Hong Kong_IHB86
Raymondia sp. B_Hong Kong_LHB14
Musca domestica
1
0.99
0.80
0.99
1
1
0.58
0.61
0.94
0.62
1
0.68
1
0.99
0.90
0.99
0.99
0.58
1
0.65
1
0.79
0.97
1
0.57
1
0.97
0.95
1
0.61
1
1
0.78
0.98
1
0.99
0.89
0.56
1
1
1
1
1
0.4

b)

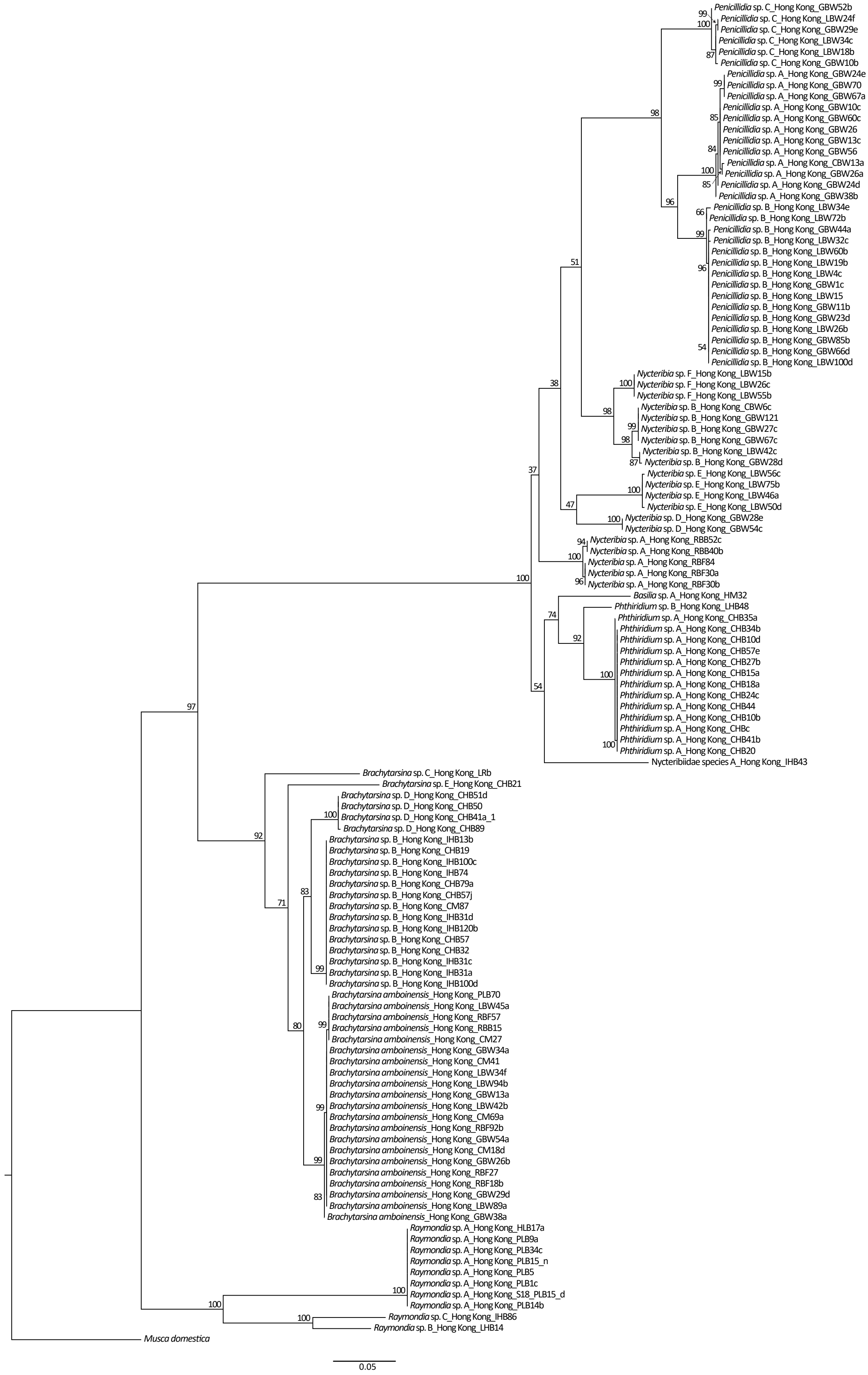

Penicillidia sp. C_Hong Kong_GBW52b
Penicillidia sp. C_Hong Kong_LBW24f
Penicillidia sp. C_Hong Kong_GBW29e
Penicillidia sp. C_Hong Kong_LBW34c
Penicillidia sp. C_Hong Kong_LBW18b
Penicillidia sp. C_Hong Kong_GBW10b
Penicillidia sp. A_Hong Kong_GBW24e
Penicillidia sp. A_Hong Kong_GBW70
Penicillidia sp. A_Hong Kong_GBW67a
Penicillidia sp. A_Hong Kong_GBW10c
Penicillidia sp. A_Hong Kong_GBW60c
Penicillidia sp. A_Hong Kong_GBW26
Penicillidia sp. A_Hong Kong_GBW13c
Penicillidia sp. A_Hong Kong_GBW56
Penicillidia sp. A_Hong Kong_CBW13a
Penicillidia sp. A_Hong Kong_GBW26a
Penicillidia sp. A_Hong Kong_GBW24d
Penicillidia sp. A_Hong Kong_GBW38b
Penicillidia sp. B_Hong Kong_LBW34e
Penicillidia sp. B_Hong Kong_LBW72b
Penicillidia sp. B_Hong Kong_GBW44a
Penicillidia sp. B_Hong Kong_LBW32c
Penicillidia sp. B_Hong Kong_LBW60b
Penicillidia sp. B_Hong Kong_LBW19b
Penicillidia sp. B_Hong Kong_LBW4c
Penicillidia sp. B_Hong Kong_GBW1c
Penicillidia sp. B_Hong Kong_LBW15
Penicillidia sp. B_Hong Kong_GBW11b
Penicillidia sp. B_Hong Kong_GBW23d
Penicillidia sp. B_Hong Kong_LBW26b
Penicillidia sp. B_Hong Kong_GBW85b
Penicillidia sp. B_Hong Kong_GBW66d
Penicillidia sp. B_Hong Kong_LBW100d
Nycteribia sp. F_Hong Kong_LBW15b
Nycteribia sp. F_Hong Kong_LBW26c
Nycteribia sp. F_Hong Kong_LBW55b
Nycteribia sp. B_Hong Kong_CBW6c
Nycteribia sp. B_Hong Kong_GBW121
Nycteribia sp. B_Hong Kong_GBW27c
Nycteribia sp. B_Hong Kong_GBW67c
Nycteribia sp. B_Hong Kong_LBW42c
Nycteribia sp. B_Hong Kong_GBW28d
Nycteribia sp. E_Hong Kong_LBW56c
Nycteribia sp. E_Hong Kong_LBW75b
Nycteribia sp. E_Hong Kong_LBW46a
Nycteribia sp. E_Hong Kong_LBW50d
Nycteribia sp. D_Hong Kong_GBW28e
Nycteribia sp. D_Hong Kong_GBW54c
Nycteribia sp. A_Hong Kong_RBB52c
Nycteribia sp. A_Hong Kong_RBB40b
Nycteribia sp. A_Hong Kong_RBF84
Nycteribia sp. A_Hong Kong_RBF30a
Nycteribia sp. A_Hong Kong_RBF30b
Basilia sp. A_Hong Kong_HM32
Phthiridium sp. B_Hong Kong_LHB48
Phthiridium sp. A_Hong Kong_CHB35a
Phthiridium sp. A_Hong Kong_CHB34b
Phthiridium sp. A_Hong Kong_CHB10d
Phthiridium sp. A_Hong Kong_CHB57e
Phthiridium sp. A_Hong Kong_CHB27b
Phthiridium sp. A_Hong Kong_CHB15a
Phthiridium sp. A_Hong Kong_CHB18a
Phthiridium sp. A_Hong Kong_CHB24c
Phthiridium sp. A_Hong Kong_CHB44
Phthiridium sp. A_Hong Kong_CHB10b
Phthiridium sp. A_Hong Kong_CHBc
Phthiridium sp. A_Hong Kong_CHB41b
Phthiridium sp. A_Hong Kong_CHB20
Nycteribiidae species A_Hong Kong_IHB43
Brachytarsina sp. C_Hong Kong_LRb
Brachytarsina sp. E_Hong Kong_CHB21
Brachytarsina sp. D_Hong Kong_CHB51d
Brachytarsina sp. D_Hong Kong_CHB50
Brachytarsina sp. D_Hong Kong_CHB41a_1
Brachytarsina sp. D_Hong Kong_CHB89
Brachytarsina sp. B_Hong Kong_IHB13b
Brachytarsina sp. B_Hong Kong_CHB19
Brachytarsina sp. B_Hong Kong_IHB100c
Brachytarsina sp. B_Hong Kong_IHB74
Brachytarsina sp. B_Hong Kong_CHB79a
Brachytarsina sp. B_Hong Kong_CHB57j
Brachytarsina sp. B_Hong Kong_CM87
Brachytarsina sp. B_Hong Kong_IHB31d
Brachytarsina sp. B_Hong Kong_IHB120b
Brachytarsina sp. B_Hong Kong_CHB57
Brachytarsina sp. B_Hong Kong_CHB32
Brachytarsina sp. B_Hong Kong_IHB31c
Brachytarsina sp. B_Hong Kong_IHB31a
Brachytarsina sp. B_Hong Kong_IHB100d
Brachytarsina amboinensis_Hong Kong_PLB70
Brachytarsina amboinensis_Hong Kong_LBW45a
Brachytarsina amboinensis_Hong Kong_RBF57
Brachytarsina amboinensis_Hong Kong_RBB15
Brachytarsina amboinensis_Hong Kong_CM27
Brachytarsina amboinensis_Hong Kong_GBW34a
Brachytarsina amboinensis_Hong Kong_CM41
Brachytarsina amboinensis_Hong Kong_LBW34f
Brachytarsina amboinensis_Hong Kong_LBW94b
Brachytarsina amboinensis_Hong Kong_GBW13a
Brachytarsina amboinensis_Hong Kong_LBW42b
Brachytarsina amboinensis_Hong Kong_CM69a
Brachytarsina amboinensis_Hong Kong_RBF92b
Brachytarsina amboinensis_Hong Kong_GBW54a
Brachytarsina amboinensis_Hong Kong_CM18d
Brachytarsina amboinensis_Hong Kong_GBW26b
Brachytarsina amboinensis_Hong Kong_RBF27
Brachytarsina amboinensis_Hong Kong_RBF18b
Brachytarsina amboinensis_Hong Kong_GBW29d
Brachytarsina amboinensis_Hong Kong_LBW89a
Brachytarsina amboinensis_Hong Kong_GBW38a
Raymondia sp. A_Hong Kong_HLB17a
Raymondia sp. A_Hong Kong_PLB9a
Raymondia sp. A_Hong Kong_PLB34c
Raymondia sp. A_Hong Kong_PLB15_n
Raymondia sp. A_Hong Kong_PLB5
Raymondia sp. A_Hong Kong_PLB1c
Raymondia sp. A_Hong Kong_S18_PLB15_d
Raymondia sp. A_Hong Kong_PLB14b
Raymondia sp. C_Hong Kong_IHB86
Raymondia sp. B_Hong Kong_LHB14
Musca domestica
0.05

Figure S21 Phylogenetic relationships of all DNA barcoded individuals of the 20 bat fly species identified in this study, inferred from the a) Bayesian inference (BI) and b) maximum likelihood (ML) methods based on the *COI* gene (609bp). The values before each node represent the Bayesian posterior probability and the ML bootstrap value.
